# Study on the gut symbiotic microbiota in long- and short-winged brown planthopper, *Nilaparvata lugens* (Stål)

**DOI:** 10.1038/s41598-024-62350-2

**Published:** 2024-05-17

**Authors:** Jingjing Zhao, Guangxiang Guan, Danting Li, Xiaoping Yu, Xuping Shentu

**Affiliations:** Zhejiang Provincial Key Laboratory of Biometrology and Inspection and Quarantine, College of Life Science, China Jiliang University, Hangzhou, 310018 China

**Keywords:** *Nilaparvata lugens*, Wing dimorphism, Gut symbiotic microbiota, Symbiotic bacteria diversity, High throughput sequencing, Biochemistry, Microbiology, Molecular biology

## Abstract

The brown planthopper (BPH), *Nilaparvata lugens* (Stål), is one of the most important rice pests in Asia rice regions. BPH has monophagy, migration, rapid reproduction and strong environmental adaptability, and its control is a major problem in pest management. Adult BPH exhibit wing dimorphism, and the symbiotic microbiota enriched in the gut can provide energy for wing flight muscles as a source of nutrition. In order to study the diversity of symbiotic microbiota in different winged BPHs, this paper takes female BPH as the research object. It was found that the number of symbiotic microbiota of different winged BPHs would change at different development stages. Then, based on the 16S rRNA and ITS sequences, a metagenomic library was constructed, combined with fluorescent quantitative PCR and high-throughput sequencing, the dominant symbiotic microbiota flora in the gut of different winged BPHs was found, and the community structure and composition of symbiotic microbiota in different winged BPHs were further determined. Together, our results preliminarily revealed that symbiotic microbiota in the gut of BPHs have certain effects on wing morphology, and understanding the mechanisms underlying wing morph differentiation will clarify how nutritional factors or environmental cues alter or regulate physiological and metabolic pathways. These findings also establish a theoretical basis for subsequent explorations into BPH-symbiont interplay.

## Introduction

The brown planthopper (BPH), *Nilaparvata lugens* (Stål) (Hemiptera: Delphacidae), is a significant monophagous herbivore of rice in Asia, which causes harms to rice by feeding on the phloem of rice plants and transmitting virus^1^. The BPH has led to substantial losses to China’s agricultural economy. Through long-term coevolution with its host, BPH has developed symbiotic associations with gut microbes that provide key nutritional and physiological benefits. BPH supplies a stable intra-host environment for its microbiota and they share certain metabolic pathways, while gut symbionts can use the metabolic nitrogen waste of the host to synthesize essential amino acid, thus providing the host with nutrients that are lacking in the food. BPH-symbiont relationships have additionally been shown to enhance host immunity, influence behavioral traits, mediate insecticide resistance and detoxification^2–4^. Comprehensive analysis of the symbiont species diversity across BPH wing morphs is therefore critical for elucidating the contributions of gut bacteria to insect development, ecology, and pest status. Characterization of symbiont diversity and composition will establish a foundation for future investigations into microbe-insect interactions affecting BPH physiology and life history.

Adult BPH exhibit wing dimorphism, this represents a strategy between insects’ flight stability and reproductive capacity. Numerous studies indicate that wing dimorphic insects are well-suited subjects for investigating the evolution of dispersal and fecundity. One is the long-winged (macropterous) which is easy to migrate and spread but has low reproductive ability, and the other is the short (brachypterous) which is easy to reproduce but has no flight ability^5–7^. Symbiotic microbiota within insects share an intimate symbiotic relationship with their hosts, primarily inhabiting locations such as the intestinal tract. The host’s physiological and biochemical fluctuations influence the symbiont populations. The insect gut microbiota closely relates to insect digestion, nutritional provision, pesticide resistance mechanisms, and host physiological function^8^. Symbionts of BPHs can utilize the host’s metabolic nitrogenous waste to synthesize essential amino acids requisite for BPHs growth and development, impacting the host’s reproductive capacity, pesticide resistance, and detoxification abilities^9–12^. The investigations delineating the symbiont populations within long- and short-winged BPHs have yet to be documented, and it is not clear which symbiotic microbiota in the gut differ among different winged insects. Although it is generally believed that both genetic and environmental factors affect the development of insect wing types, for example, there are differences in the community structure and diversity of symbiotic microbiota in BPHs that feed on different resistant rice and are at different developmental stages; the diversity of symbiotic microbiota in the gut of male adults of BPHs is the most abundant, while the diversity of 5th instar nymphs is the poorest. Microbial community structure also exerts critical influence over the internal environmental system of insects, constituting an indispensable parameter for illuminating insect internal environments. Insect gut microbes have certain impacts upon host physiological functions^13–17^, with symbiotic bacteria capable of modulating host metabolism, facilitating efficient digestion and nutrient acquisition, and conferring protection against pathogenic microorganisms; Symbiotic fungi exhibit potential biocontrol capacities while exhibiting intricate population structures and complex multipartite interactions^18,19^. Among insects, fungi are also an important food source for insects because they can accumulate large amounts of nitrogen, phosphorus and organic compounds, such as chitin^20^. Numerous symbiotic fungi can biosynthesize amino acids absent from their insect hosts’ diets^21^.

In this study, based on the intimate symbiotic relationship between BPHs and microorganisms, we utilized different winged female BPHs as model research subjects, and through techniques including fluorescent quantitative PCR and high-throughput sequencing, we quantified changes in symbiont abundance across wing morphs and developmental timepoints. Additionally, we analyzed the differences in community diversity and populations of symbionts in the gut of different winged BPHs, and made a preliminary prediction of the functions of differential symbionts, as well as explored the relationship between the different winged BPHs and the community structure of symbionts. These efforts not only enrich the knowledge of insect wing morphs and symbiotic microbiota, but also lay a theoretical foundation for the subsequent research of BPH-symbiotic microbiota interaction and the biological function of insect symbiotic microbiota. In summary, elucidating the gut microbiome dynamics associated with host wing dimorphism constitutes a critical undertaking to clarify poorly understood facets of insect symbioses, with potential implications for informing pest management approaches.

## Materials and methods

### Source and feeding of brown planthopper

Rice variety: The rice variety used in this experiment was Taichung Native 1 (TN1), a susceptible cultivar, which was obtained from the International Rice Research Institute in 2008 and were subsequently cultivated and used continuously in our laboratory. TN1 seeds were soaked in water until pale in coloration. The seeds were then evenly distributed in acrylic trays containing perlite substrate and grown to the three-leaf stage under controlled environmental conditions. TN1 seedlings were used to feed the test insects.

Test insects: The BPH used in this study was collected from the rice field (E120 °12′, N30 °16′) in Hangzhou, Zhejiang Province, China. The susceptible rice variety TN1 was reared in an artificial climate chamber (26 ± 1 ℃, L:D = 16 h:8 h, relative humidity 65–75%) for a long time and has been continuously cultured for more than ten generations under constant feeding conditions. After the BPH eclosion to adult, the long-winged female BPHs and the short- winged female BPHs were collected and placed in 500 mL plastic cups with three-leaf rice seedlings, and then brought 150 different winged BPHs back to the laboratory for dissection, respectively. A total of three biological repetitions.

### Preparation of standard plasmids and construction of standard curves

Preparation of standard plasmids: Using the TaKaRa MiniBEST Universal RNA Extraction Kit to extract the mRNA genome of BPHs and reverse transcribed to synthesize cDNA with the PrimeScript™ RT reagent Kit with gDNA Eraser; and ordinary PCR was performed using Nlβ-Actin primers (Nlβ-ActinF: 5′-GATGAGGCGCAGTCAAAGAG-3′, Nlβ-ActinR: 5′-GTCATCTTCTCACGGTTGGC-3′) and *N. lugens* cDNA as template to amplify the β-Actin sequence; The V3–V4 region was amplified via Colony PCR employing primers 338F (5′-ACTCCTACGGGAGGCAGCA-3′) and 806R (5′-GGACTACHVGGGTWTCTAAT-3’) and using *Escherichia coli* (*E. coli*) as template; The ITS2 sequence was amplified by Colony PCR utilizing primers ITS3F (5′-GCATCGATGAAGAACGCAGC-3′) and ITS4R (5′-TCCTCCGCTTATTGATATGC-3′) and *Saccharomyces cerevisiae* (*S. cerevisiae*) as template. Agarose gel electrophoresis and gel imaging were utilized to confirm expected band sizes for the three PCR products, and the correct bands were recovered by gel purification. The recovered amplicons were ligated into pMD18-T vectors and incubated at 16 °C for 3 h, and the ligation products were transformed into JM109 competent cells, evenly plated on LB (Luria–Bertani) solid medium containing 100 μg/mL ampicillin, and incubated inverted overnight at 37 °C. Then pick single colonies from the above plate and inoculate into 1 mL LB liquid medium containing 100 μg/mL ampicillin at 180 rpm, 37 °C for 4 h. Colony PCR was performed to validate the above cultures, and positive clones with correct validation were selected to prepare standard plasmids.

Construction of standard curves: Using the SanPrep Column Plasmid DNA Mini-Extraction Kit to extract the plasmids and Nanodrop microspectrophotometry to assess the purity and concentration of the plasmid DNA, then calculating the plasmid copy number. And the plasmid DNA was diluted in a tenfold gradient to make 10^10^–10^6^ copies/μL of the product. The plasmid DNA templates at each dilution were subjected to fluorescent quantitative PCR under the specified conditions. The constructed standard plasmids were then utilized for fluorescent quantitative PCR of dissected gut samples from different winged BPHs which in different developmental times.Each treatment had 3 replicates. PCR reaction solution consists of 10 μL 2 × TB Green Premix Ex Taq II (Tli RNaseH Plus), 0.4 μL 10 μM Forward Primer, 0.4 μL 10 μM Reverse Primer, 2 μL Template DNA and adjusted to the final volume of 20 μL with ddH_2_O. The thermal cycling program is as follows: initial denaturation at 95 °C for 30 s; followed by 40 cycles of denaturation at 95 °C for 5 s, primer annealing at 55 °C for 30 s, and extension at 72 °C for 30 s to synthesize DNA. The standard curves were generated by plotting the logarithm of different starting copy numbers on the x-axis against the initial cycle number Ct of the fluorescent signal on the y-axis.

### Gut collection and DNA preparation of different winged BPHs

The female long- and short-winged BPHs were collected on the 1st, 2nd and 4th day after eclosion and dissected under sterile conditions: the epidermis of BPH were washed twice in 75% ethanol for 30 s, and the sterilized PBS buffer (0.01 M, pH = 7.4) was filtered for three times. After that, the BPH were immersed in sterile PBS buffer (0.01 M, pH = 7.4) solution under stereomicroscope and dissected with sterile medical anatomical tweezers and the gut of BPH was collected into 1.5 mL sterile centrifuge tube and stored in − 80 ℃ ultra-low temperature refrigerator for sequencing.

FastDNA SPIN kit (Soil Genomic DNA Extraction Kit) was used to extract DNA (OMEGA, Bio-Tek, Georgia, United States) according to the manufacturer’s protocol. DNA concentration and purity were quantified by NanoDrop 2000 spectrophotometry (Thermo Fisher Scientific, United States) and 1% agarose gel electrophoresis. All DNA samples should be preserved at − 20 °C prior to downstream analysis.

### PCR Amplification and high-throughput sequencing

The collected intestinal samples of BPH were sent to Shanghai Meiji Biomedical Technology Co., Ltd. through the Illumina Miseq platform. The V3–V4 hypervariable region of the bacterial 16S rRNA gene was amplified using primers 338F (5′-ACTCCTACGGGAGGCAGCA-3′) and 806R (5′-GGACTACHVGGGTWTCTAAT-3′) ; The ITS of the fungal ribosomal operon was amplified with universal primers ITS3F (5′-GCATCGATGAAGAACGCAGC-3′) and ITS4R (5′-TCCTCCGCTTATTGATATGC-3′) for fungal diversity analysis.

PCR reaction solution consists of 4 μL 5 × FastPfu buffer, 2 μL 2.5 mM dNTP, 0.4 μL FastPfu polymerase, 0.2 μL BSA, 10 ng template DNA and primers, and adjusted to the final volume of 20 μL with ddH_2_O. The thermal cycling program was as follows: initial denaturation at 95 °C for 3 min, followed by 27 cycles of denaturation at 95 °C for 30 s, primer annealing at 55 °C for 30 s, and extension at 72 °C for 45 s to synthesize DNA, finally, the product was kept at 72 °C for 10 min to extend completely. Before high-throughput sequencing, the concentration of all purified PCR amplifications was examined by 2% agarose gel electrophoresis and quantified by NanoDrop 2000. Then, 10 ng of 16 s rRNA gene amplification products and 10 ng of ITS gene amplification products were collected for MISEQ sequencing in MajorBio Techonology Co., Ltd., Shanghai, China.

### Bioinformatics and statistical analysis

Raw sequencing reads were processed in Qiime (v1.9.1) for quality control, filtering, splicing, chimera removal, and sequence optimization. The operational taxonomic units (OTU) clustering was performed on the optimized sequence by Uparse (v7.1) software, and the recognition threshold was 97%. RDP Classifier (v2.2) Bayesian algorithm and Qiime (v1.9.1) software were used for species comparison annotation. The default confidence threshold was 0.7. The bacterial comparison database was Silva (v132), and the fungal comparison database was Unite (v7.2). The microbial community structure was statistically analyzed at the level of boundary, phylum, class, order, family, genus and species^22^. On the basis of the above analysis, the Alpha/Beta diversity indexes such as Simpson index and Shannon index of microorganisms were calculated and analyzed by Mothur (v.1.30.1) software^23^. The larger Shannon index indicates a higher species richness, while the higher Simpson index indicates a larger composition of specific species. Alpha diversity indices and species differences between groups were compared using the Welch T test. *P* < 0.05 was considered statistically significant. PICRUSt analysis tool was used to predict bacterial community function based on 16S rRNA high-throughput sequencing. According to the high-throughput sequencing results of fungal rRNA ITS2 region, FUNGuild (Fungi Functional Guild) was used to predict the function of fungal community. The number of dominant symbiotic microbiota in the gut of different winged BPHs was obtained. The UPARSE pipeline from USEARCH was used for statistical analysis and high-quality sequences were clustered into operational taxonomic units (OTUs) with 97% similarity. Representative OTUs determined according to the highest relative abundance in each OTU were selected to construct the entire OTU table. Species richness was estimated using ACE and a non-parametric richness estimator (Chao1) based on single-photon and two-photon distributions. Graphpad Prism 8.0 was used.

### Microbial abundance detection

The total copy number of bacterial and fungal communities was calculated by qPCR. The absolute abundance of each strain was estimated based on relative abundance. The primers used in qPCR were the same as the sequencing primers and were tested in three parallel samples using TB Green Premix Ex Taq (Takara, Dalian, China), 0.4 μL 10 μM primer, 0.4 μL ROX reference dye, 6.8 μL H_2_O, and 2 μL Template DNA. PCR conditions: initial denaturation at 95 °C for 30 s; then in the real-time PCR system at 95 °C for 5 s; 40 PCR cycles were performed at 60 °C for 30 s. The absolute copy number was calculated based on the standard curve produced by amplification of target gene in the pMD18-T vector (The relevant data in this article have been published on the website of Meiji Biology) (Sangon Biotech, Shanghai, China).

### Compliance with ethics guidelines

This animal subjects and plant used for experimental studies in this article comply with the IUCN Policy Statement on Research Involving Species at Risk of Extinction and the Convention on the Trade in Endangered Species of Wild Fauna and Flora.

## Results

### The plasmids standard curves

**Figure 1:**
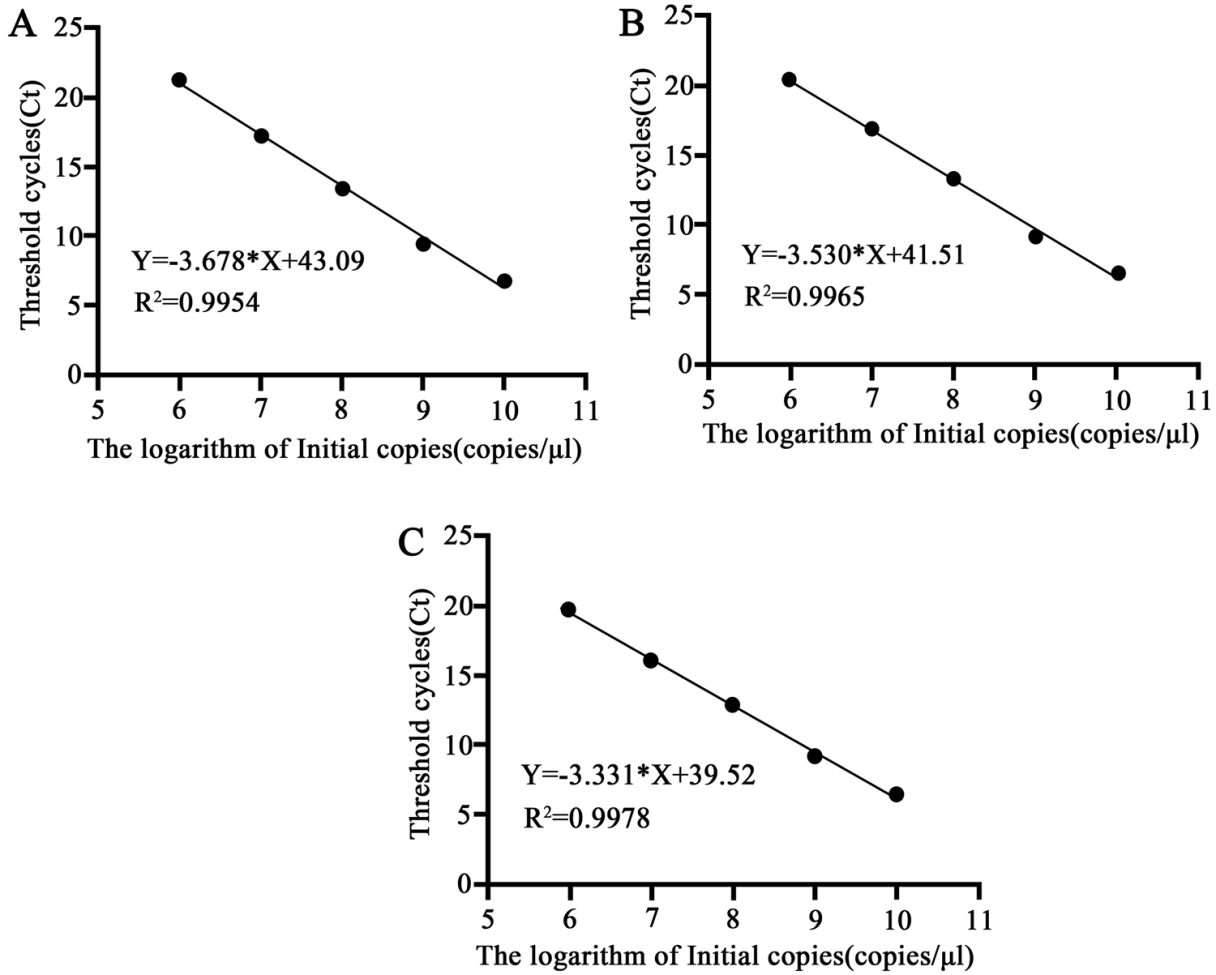
(**A**) Standard curves of three plasmids. *E. coli* 16S; (**B**) *S. cerevisiae* ITS; (**C**) *N. lugens* β-actin.

The standard curves for *E. coli*, *S. cerevisiae*, and *N. lugens* β-actin plasmids exhibited linearity with *R*^*2*^ greater than 0.99, and the amplification efficiencies ranging from 95 to 105% (Fig. 1). It shows that the reaction conditions and standard curves can be used to detect the changes in the number of symbiotic microbiota of different winged BPHs at different developmental stages of the host.

### The variation of the number of gut symbionts in different developmental stages of different winged BPHs

**Figure 2:**
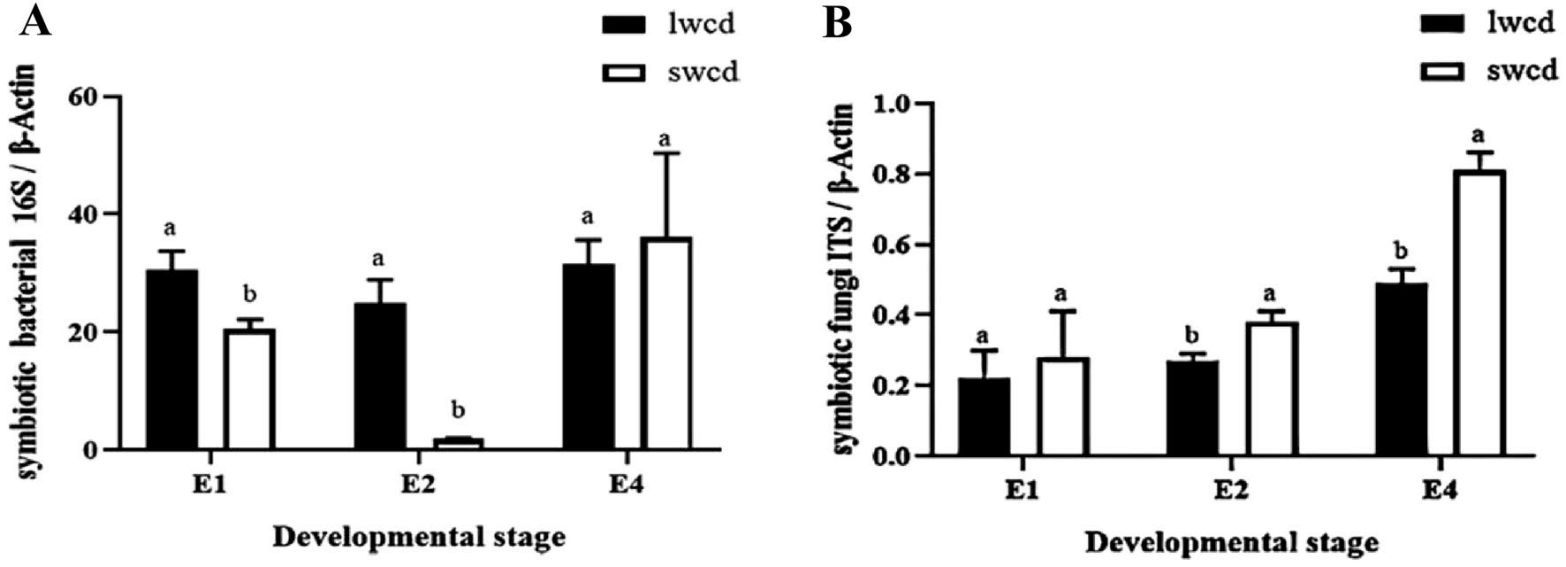
(**A**) Number of gut bacteria of different winged BPHs at different developmental stages. (**B**) Number of gut fungi of different winged BPHs at different developmental stages. E1 represents the female of BPH eclosion on the 1st day, and E2 and E4 represent the females on the 2nd and 4th days of eclosion. The ordinate is the ratio of the copy number corresponding to the ITS sequence value of the gut symbiotic microbiota to the Ct value of the β-Actin sequence of the corresponding sample. Values are mean ± SE, where n = 5. Different letters on the top of each bar indicate that the data are significantly different by Tukey test for multiple comparisons with a significance threshold of *p* < 0.05. lwcd represents the gut samples of long-winged BPH ; swcd represents the gut samples of short-winged BPH.

Quantification of the total symbiont populations in different developmental stages of different winged BPHs revealed dynamic changes during development (Fig. 2). The trend in symbiotic bacteria per gut unit decreased initially followed by an increase from the 1st day to the 4th day of emergence. There were significant differences on the 1st and the 2nd day of emergence among different winged BPHs (*P* < 0.05), the number of symbiotic bacteria per gut unit of long-winged BPH was significantly higher than that of short-winged BPH. While there was no significant differences occurred in symbiotic bacteria per gut unit on the 4th day of emergence (Fig. 2A). The trend in symbiotic fungi per gut unit exhibited a gradual increase from the 1st day to the 4th day of emergence, especially in the adult stage. There was no significant difference in the number of gut symbiotic fungi per unit of different winged BPHs on the 1st day after emergence, but there was a significant difference on the 2nd and 4th day (*P* < 0.05), the number of intestinal symbiotic fungi per unit of short-winged BPH was greatly higher than that of long-winged BPH (Fig. 2B).

### OTU clustering and annotation of gut symbiotic microbionts of different winged BPHs

**Figure 3:**
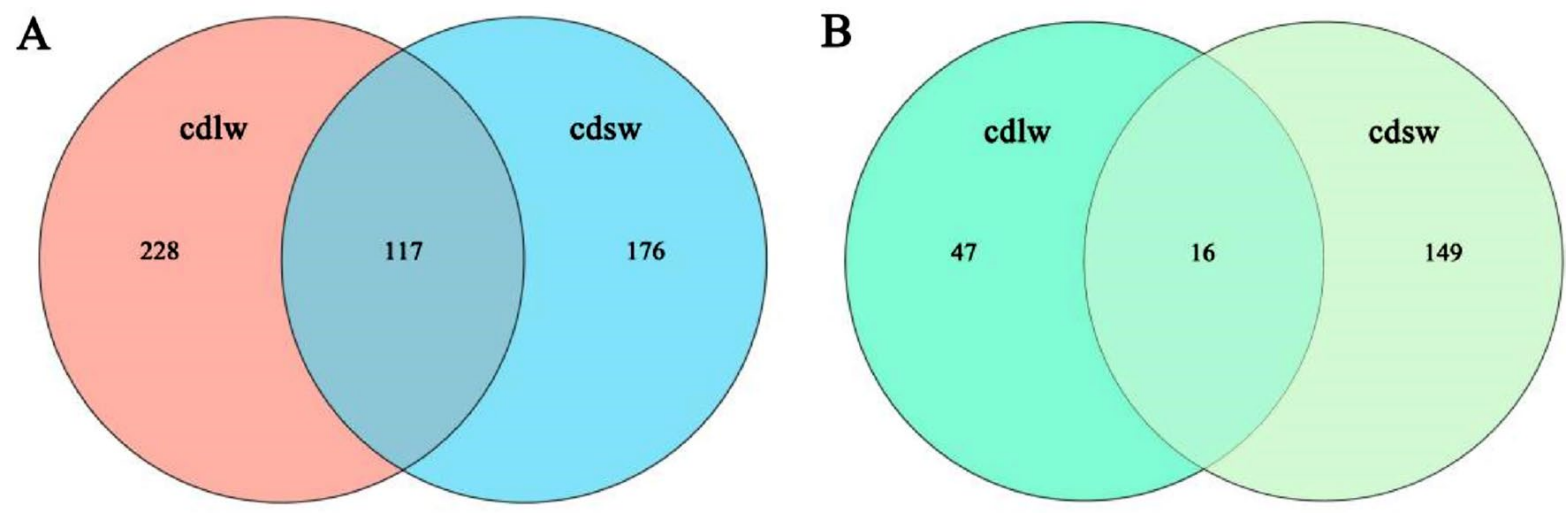
(**A**) Veen diagram of gut bacterial OTUs of different winged BPHs; (**B**) Veen diagram of gut fungi OTU of different winged BPHs; cdlw represents the gut samples of long-winged BPH; cdsw represents the gut samples of short-winged BPH.

Based on data filtering and removal of low-quality sequences, the sequencing of the bacterial 16S rRNA V3–V4 region and fungal rRNA ITS2 region of the gut samples of long- and short-winged BPHs in this experiment, the number of taxonomic status of phylum, class, order, family and genus, and the results are shown in Table 1. Select the OTU list at the genus level, and analyze the clustering results of the OTUs at the level of 97% similarity. The Veen diagram of the community structure of the symbionts in different parts of different winged BPHs in Fig. 3.

**Table 1:** OTU clustering and number of taxonomic status of gut symbiotic microbiota of different winged BPHs.

Sample	wing morphs	sequence number	OTU	Phylum	Class	Order	Family	Genus
Bacterial community	long-winged	140,802	345	15	30	78	137	245
	short-winged	105,102	293	16	32	75	115	184
Fungal community	long-winged	115,179	63	5	13	17	25	30
	short-winged	147,681	165	9	26	42	62	73

The Veen diagram present the differential expression and similarity between genes or gene families of different species, and it reflect the number of microbial species and the diversity of microorganisms of different winged BPHs. It can be obtained from the Fig. 3: There are 117 common species in the gut bacteria of different winged BPHs, accounting for 33.91% and 39.93% of the total number of species in the gut samples of long-winged BPH and short-winged BPH, respectively. The number of unique bacterial species in the gut of long-winged BPH was 228, which was higher than that of short-winged BPH (Fig. 3A). This indicates greater bacterial diversity in long-winged BPH. In contrast, 16 shared fungal species in the gut of different winged BPHs, accounting for 25.40% and 9.70% of the total number of species in the gut samples of long-winged BPH and short-winged BPH, respectively. The number of unique fungal species in the gut of long-winged BPH was 47, which was higher than that of short-winged BPH (Fig. 3B). It indicates that greater fungi diversity in short-winged BPH.

### Species abundance analysis of gut symbiotic microbiota of different winged BPHs

**Figure 4:**
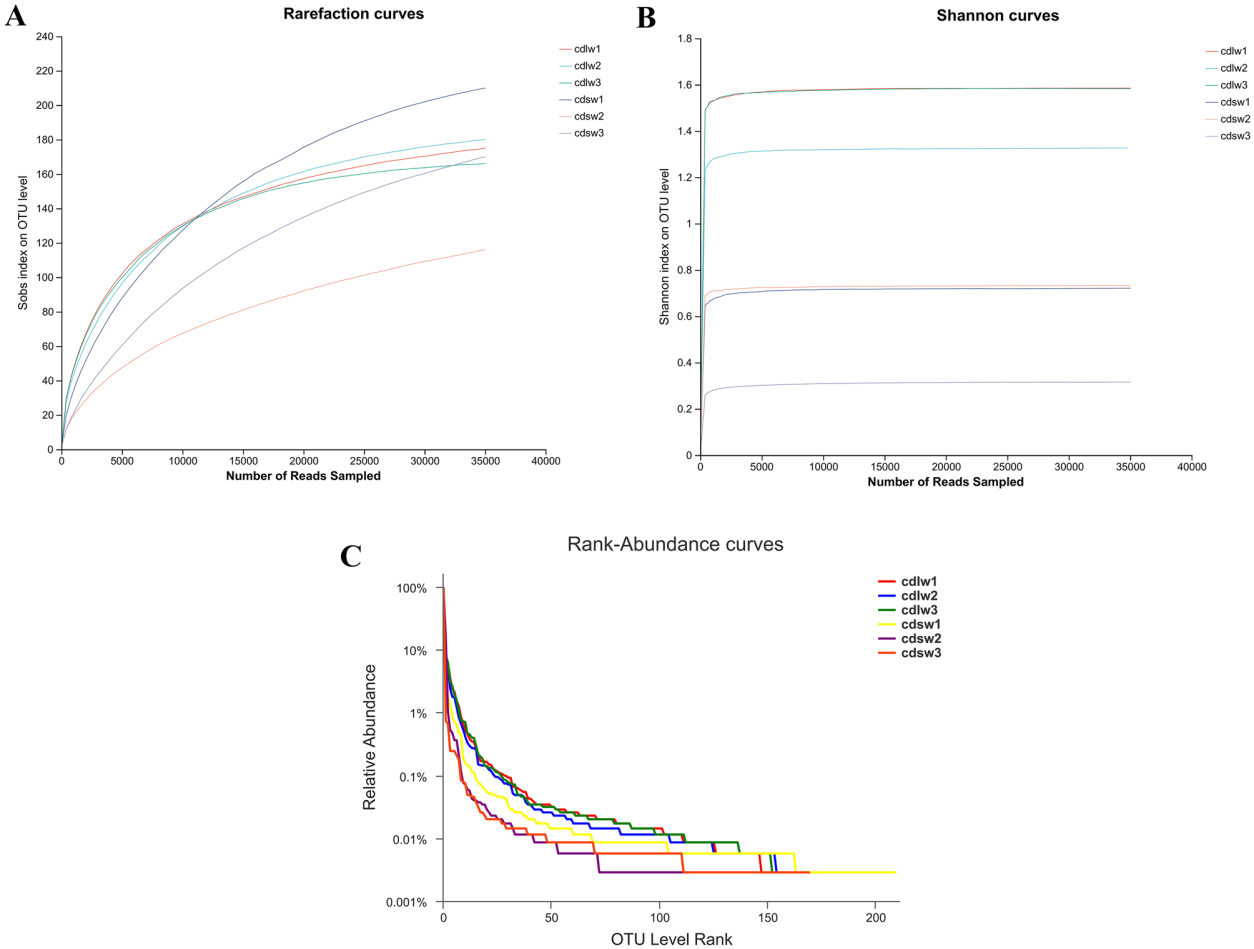
(**A**) Rarefaction curve of bacteria of different winged BPHs; (**B**) Shannon curve of bacteria of different winged BPHs; (**C**) Rank-Abundance curves of bacteria of different winged BPHs; cdlw1-3 represents three samples of the gut of long-winged BPH; cdsw1-3 represents three samples of the gut of short-winged BPH.

The species abundance of gut bacteria and fungi of different winged BPHs was analyzed (Fig. 4). The sample dilution curve shows that when the number of OTUs exceeds 10,000, the growth trend of the exponential value slows down, and the final curve tends to be horizontal (Fig. 4A), while Shannon curves plateaued at ~ 800 OTUs (Fig. 4B). This indicates sequencing depth sufficiently captured most microbial species. Bacteria species richness, denoted by curve length, was higher in short-winged BPH guts. However, community evenness, reflected in curve smoothness, was greater in long-winged BPH signifying higher diversity compared to short-winged BPH, which had a higher relative abundance of dominant bacterial population (Fig. 4C). In summary, the short-winged BPH guts exhibited greater bacterial species richness, and under a certain species richness, while long-winged BPH guts showed higher diversity with fewer dominant bacterial population.

**Figure 5:**
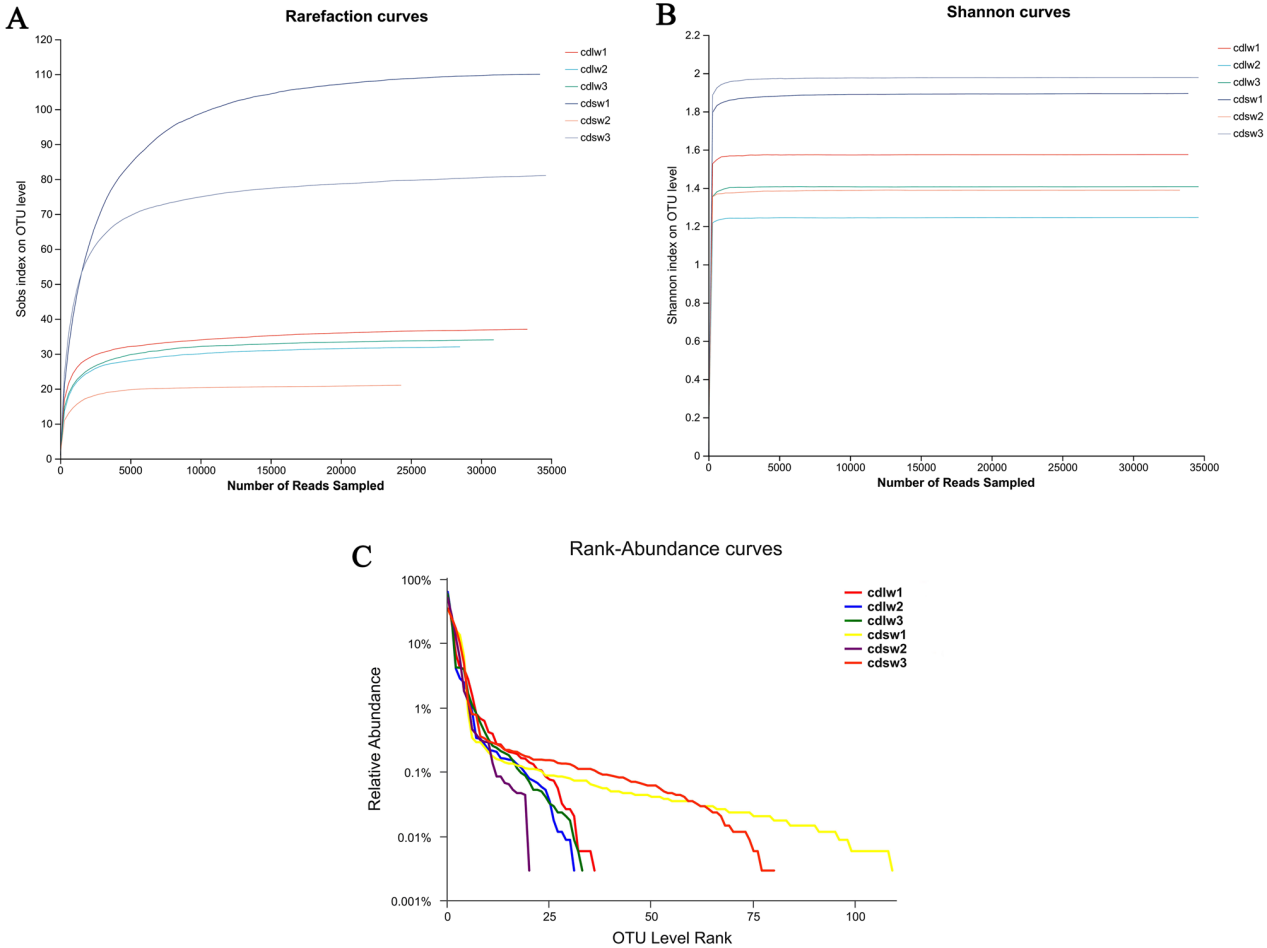
(**A**) Rarefaction curve of fungi of different winged BPHs; (**B**) Shannon curve of fungi of different winged BPHs; (**C**) Rank-Abundance curves of fungi in different winged BPHs; cdlw1-3 represents three samples of the gut of long-winged BPH; cdsw1-3 represents three samples of the gut of short-winged BPH.

Likewise, fungal diversity analysis (Fig. 5) demonstrated rarefaction curve plateaus around 5000 OTUs (Fig. 5A) and Shannon curve plateaus after ~ 300 OTUs (Fig. 5B), indicating sufficient sequencing depth to capture most microbial species. Fungal species richness, denoted by curve length, was higher in short-winged BPH guts; However, community evenness, reflected in curve smoothness, was lower in long-winged BPH guts indicating reduced diversity compared to short-winged BPH guts, which had a lower relative abundance of dominant gut fungal population (Fig. 5C). To summarize, long-winged BPHs guts exhibited lower fungal species richness and evenness, with a higher proportion of dominant fungal population compared to greater diversity in short-winged BPH guts.

### Alpha diversity analysis of gut symbiotic microbiota of different winged BPHs

Coverage values exceeded 0.99 approaching 1 across all gut samples, indicating sufficient sequencing depth to capture the all species, this aligns with the trends denoted by the Rarefaction curve and Shannon curve. The Chao index and Ace index of gut symbiotic microbiota (bacteria and fungi) in short-winged BPH were higher than those in long-winged BPH (Table 2), which showed that the abundance of symbionts in short-winged BPH was higher than that in long-winged BPH; The Shannon index was higher and Simpson index lower for long-winged BPH versus short-winged BPH bacterial communities, denoting greater diversity and evenness in gut bacteria of long-winged BPH, while the fungal samples showed the opposite trend. Comprehensive analysis of species richness and diversity information presented by the Alpha index of symbiotic microbes: The bacteria of long-winged BPH exhibited lower richness but higher diversity, while short-winged BPH showed elevated richness yet reduced diversity, reflecting high proportion of dominant bacterial population in the gut of short-winged BPH; The fungal communities of long-winged BPH displayed reduced richness and diversity, while short-winged BPH had higher richness and diversity. This indicates a greater proportion of dominant fungal species but fewer total species in the guts of long-winged BPH, denoting lower alpha diversity of the fungal communities compared to short-winged BPH.

**Table 2:** Alpha diversity index of gut symbiotic microbiota of different winged BPHs.

Sample	wing morphs	Shannon	Simpson	Ace	Chao	Coverage
Bacterial community	long-winged	1.50 ± 0.15a	0.49 ± 0.05b	185.16 ± 12.17a	184.90 ± 11.46a	0.9994 ± 0.0002
	short-winged	0.59 ± 0.24b	0.79 ± 0.13a	211.54 ± 38.07a	200.68 ± 38.51a	0.9986 ± 0.0002
Fungal community	long-winged	1.41 ± 0.16a	0.39 ± 0.05a	34.78 ± 2.72a	34.33 ± 2.52a	1.0000 ± 0.0000a
	short-winged	1.75 ± 0.32a	0.26 ± 0.07a	64.44 ± 57.44a	71.33 ± 45.63a	0.9999 ± 0.0001a

Values denote mean ± standard deviation (n = 3). Different letters within a column indicate significant differences between treatments (*P* < 0.05) based on Welch’s T-test.

### Symbiont community composition at the genus level of different winged BPHs

**Figure 6:**
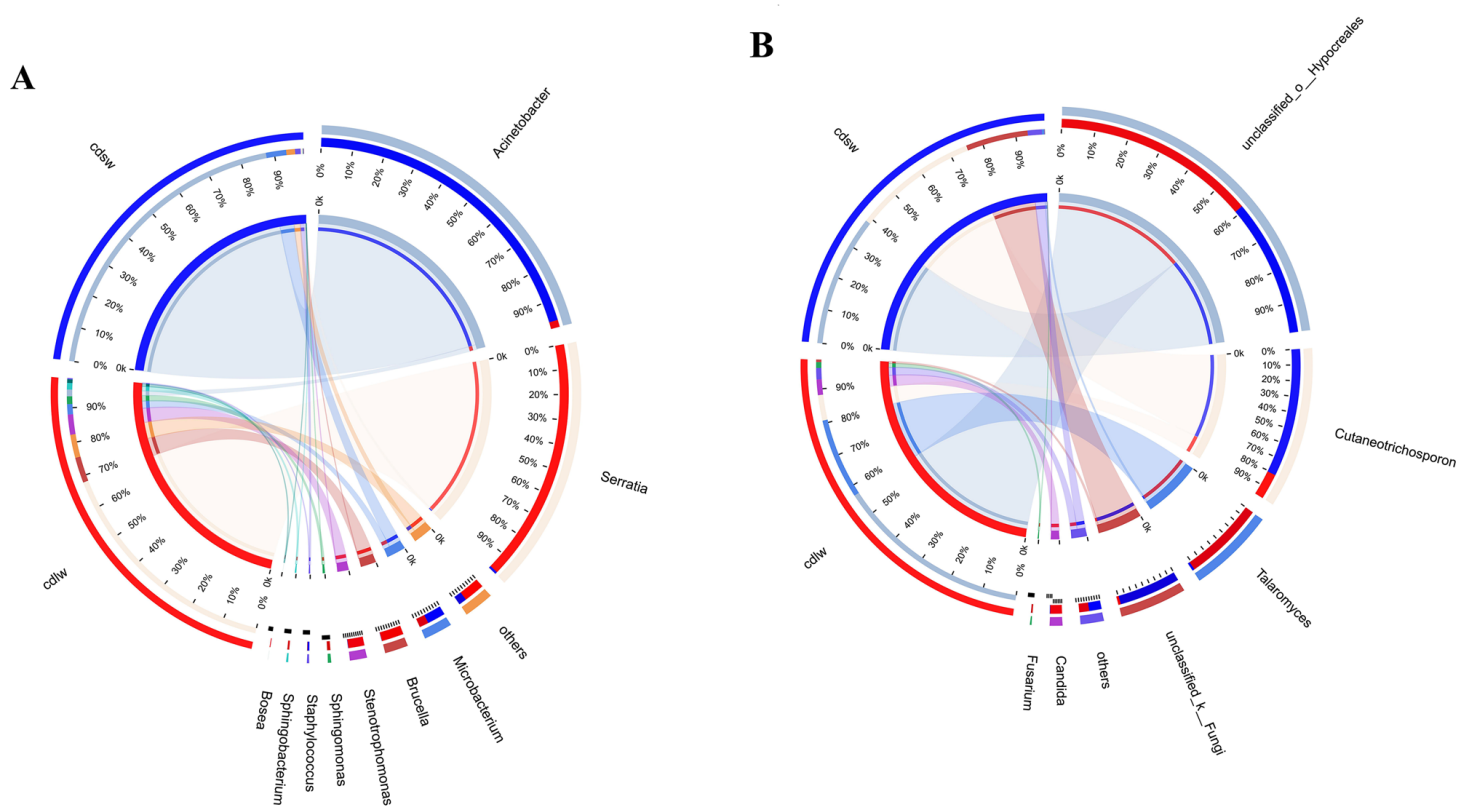
(**A**) Community composition of gut symbiotic bacteria of different winged BPHs; (**B**) Community composition of gut symbiotic fungi of different winged BPHs; cdlw represents gut samples of long-winged BPH, cdsw represents gut samples of short-winged BPH.

Figure 6A is the taxonomic stacking diagram of symbiotic bacteria with a relative abundance greater than 1% at the genus level in the gut of different winged BPHs. A total of 9 bacterial genera were detected in the V3–V4 region of the 16S rRNA gene by high-throughput sequencing. The bacterial species coexisting in the gut of the two winged BPHs mainly include: *Acinetobacter*, *Serratia*, *Microbacterium*, *Brucella*, *Stenotrophomonas*, *Sphingomonas*, *Staphylococcus*, *Sphingobacterium*, *Bosea*. Figure 6B is the taxonomic stacking diagram of symbiotic fungi with a relative abundance greater than 1% at the genus level in the gut of different winged BPHs, and 6 fungal genera were detected in the ITS2 region, with *unclassified_o_Hypocreales*, *Cutaneotrichosporon*, *Talaromyces*, *unclassified_Fungi*, *Candida*, and *Fusarium* jointly inhabiting the guts.

Although the symbiotic bacterial community structure of the long- and short-winged BPHs was similar, the overall proportion of various microorganisms was quite different. *Acinetobacter* is the dominant bacteria in the gut of short-winged BPH, accounting for 88.82%. *Serratia* is the most dominant bacterial group in the gut of long-winged BPH, accounting for 69.13% (Fig. 6A). For fungi, *Unclassified_o_Hypocreales* dominated both wing morphs, with *talaromyces* is the second dominant in the gut of long-winged BPH and *cutaneotrichosporon* is the second dominant fungus in the gut of short-winged BPH (Fig. 6B). In synopsis, it shows that the dominant bacteria in the gut of different winged BPHs are quite different, which may be related to the different biological functions.

### *Beta* diversity analysis of gut symbiotic *bacteria* of different winged BPHs

**Figure 7:**
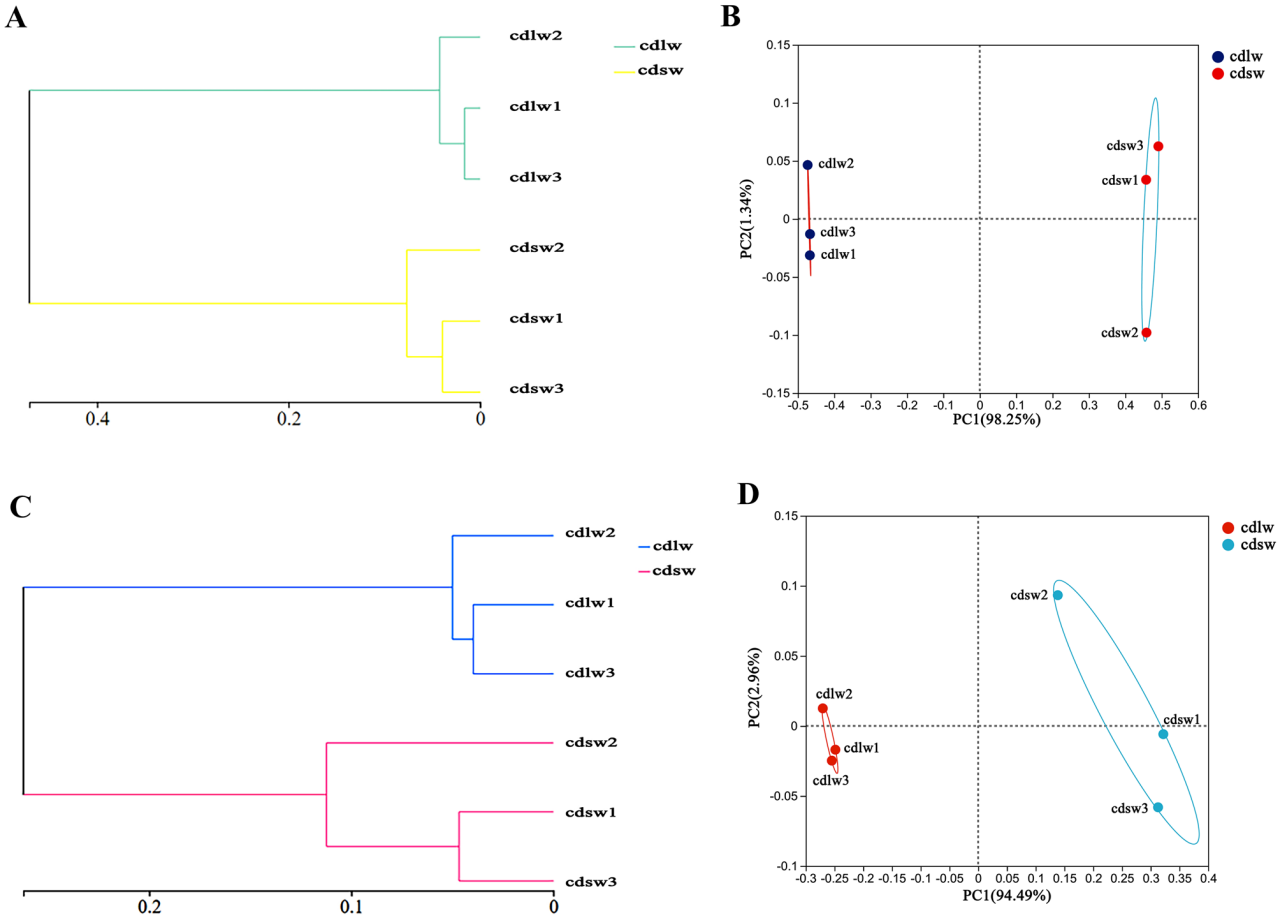
(**A**) Beta diversity analysis of gut symbiotic bacteria of different winged BPHs. The sample-level clustering diagram based on the Bray Curtis algorithm at the OTU level of the gut bacteria of different winged BPHs; (**B**) The PcoA map based on the Bray Curtis algorithm at the OTU level of the gut bacteria of different winged BPHs; (**C**) The sample-level clustering diagram based on the Bray Curtis algorithm at the OTU level of the gut fungi of different winged BPHs; (**D**) The PcoA map based on the Bray Curtis algorithm at the OTU level of the gut fungi of different winged BPHs; cdlw represents gut samples of long-winged BPH, cdsw represents gut samples of short-winged BPH.

The distance matrix of 6 samples of gut symbiotic bacteria of different long- and short-winged BPHs was obtained by Bray Curtis algorithm. Samples with proximate distances clustered into common groups, yielding hierarchical dendrograms (Fig. 7A, 7C). A non-restricted principal axis analysis (PCoA) based on Bray–Curtis distances showed that the BPHs intestinal commensal community composition clearly formed two groups, separated on the first axis, which could explain 98.25% of the differences in bacterial samples (Fig. 7B) and 94.49% of the differences in fungal samples (Fig. 7D). In summary, the wing morph is an important factor affecting the difference of intestinal bacterial community in BPHs.

### Functional prediction of gut symbiont communities of different winged BPHs

**Figure 8:**
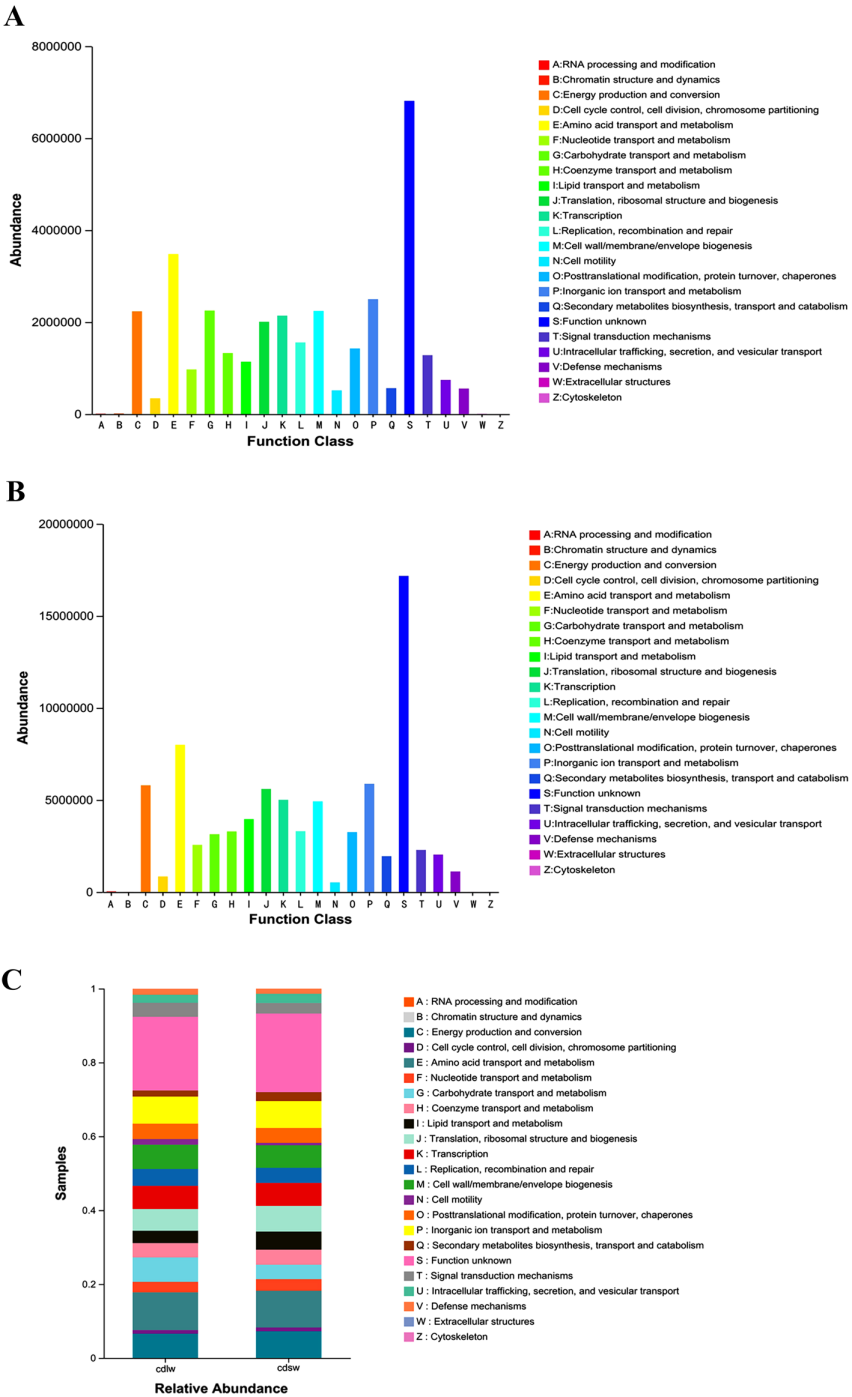
(**A**) Species of symbiotic bacteria in long-winged BPH; (**B**) Species of symbiotic bacteria in long-winged BPH; (**C**) Functional composition and abundance of gut symbiotic bacterial communities in different winged BPHs. Predictive analysis of intestinal symbiotic bacterial community function of different winged BPHs.

The COG prediction of the gut bacterial samples of BPHs is shown in Fig. 8. For the gut bacteria samples of long-winged BPH, the top 5 predicted functional abundances are amino acid transport and metabolism, inorganic ion transport and metabolism, carbohydrate transport and metabolism, cell wall/membrane/envelope biosynthesis, energy production and conversion (Fig. 8A); For the gut bacterial samples of short-winged BPH, the top 5 predicted functional abundances are amino acid transport and metabolism, energy production and conversion, translation and ribosomal structure and biotransformation, transcription, and cell wall/membrane/envelope biosynthesis, respectively (Fig. 8B); Additionally, the functions of many bacteria have not yet been clarified, which needs to be further studied in the future; The abundance of the predicted functions of short-winged BPH was mostly higher than that of long-winged BPH: Chromatin structure/dynamics and cytoskeleton functional abundances were enriched in the gut bacteria of long-winged BPH (Fig. 8C), and only the function of extracellular structure is more abundant in the gut bacteria of long-winged BPHs than in short-winged BPH (Fig. 8C). This selective enrichment may indicate that gut bacteria have a certain role in the wing morph differentiation of BPHs.

Through the analysis of the KEGG database, it was found that there are more than 2050 kinds of enzymes related to the gut bacteria of different winged BPHs. Table 3 shows the top 10 most abundant enzymes by mean value. Among them, histidine kinase and DNA-directed DNA polymerase exhibited the higher mean abundances, with consistently higher levels in short-winged BPH. Therefore, it is speculated that BPHs wing morphology may affect the composition and content of bacterial communities in the body, consequently influencing histidine kinase^24,25^ levels, potentially eliciting downstream effects on insect physiology and behavior.

**Table 3:** Abundance of major enzymes in the KEGG database of gut bacteria of different winged BPHs.

Enzyme Name	Abundance		
	cdlw	cdsw	Mean abundance
Histidine kinase	115,037.93	334,199.75	224,618.84
DNA-directed DNA polymerase	124,626.38	311,098.82	217,862.60
NADH:ubiquinone reductase (H(+)-translocating)	106,141.74	316,475.58	211,308.66
Peptidylprolyl isomerase	93,354.29	326,234.65	209,794.47
DNA helicase	117,792.25	279,908.35	198,850.30
Glutathione transferase	65,594.90	250,607.07	158,100.99
3-oxoacyl-[acyl-carrier-protein] reductase	72,855.39	134,859.55	103,857.47
3-oxoadipate CoA-transferase	13,721.24	186,231.57	99,976.41
Site-specific DNA-methyltransferase (adenine-specific)	29,323.56	159,633.88	94,478.72
Acetyl-CoA C-acetyltransferase	28,486.17	159,908.35	94,197.26

cdlw represents gut samples of long-winged BPHs, cdsw represents gut samples of short-winged BPH.

**Figure 9:**
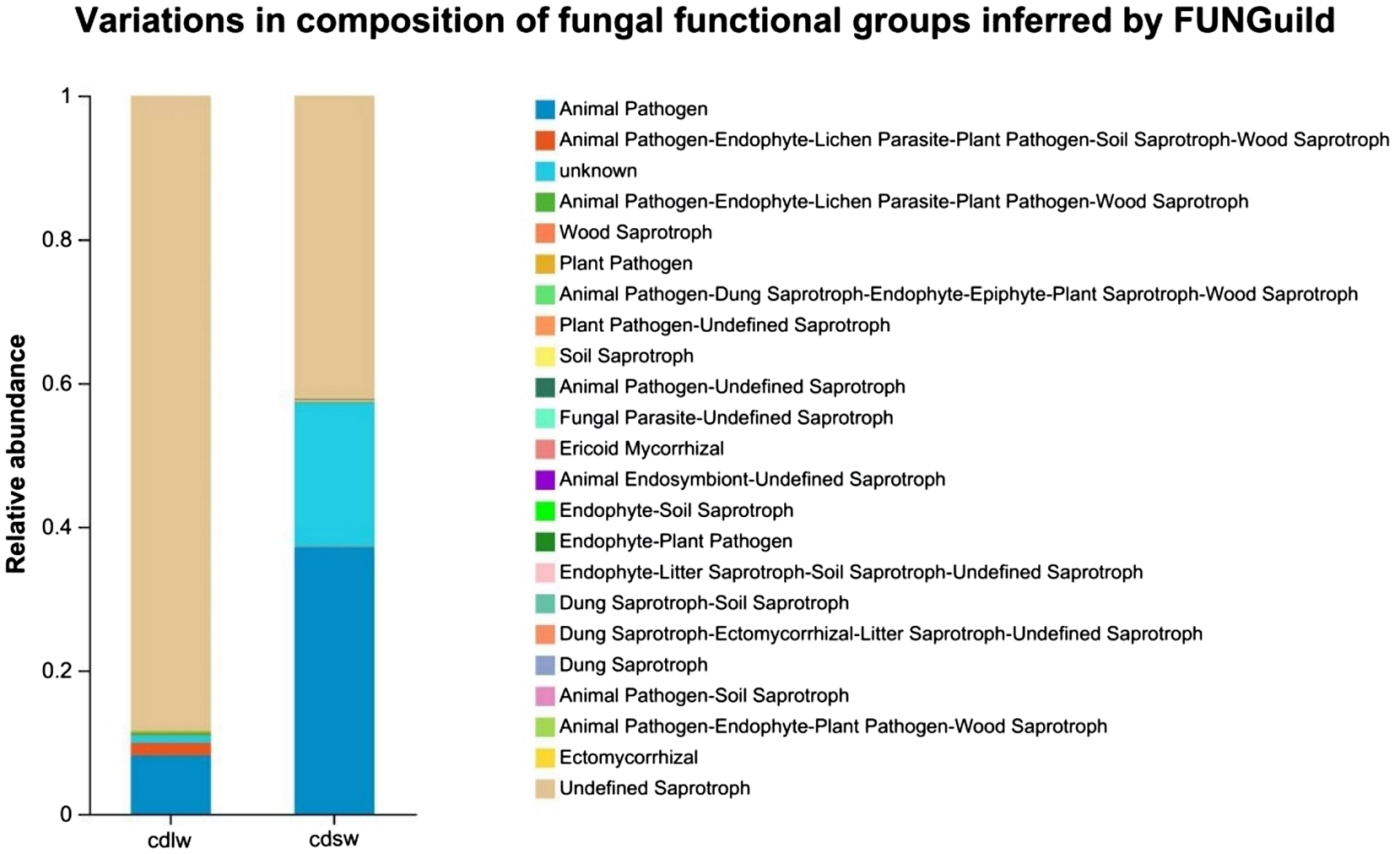
Functional composition and abundance of gut symbiotic fungi of different winged BPHs.

FUNGuild analysis classified and quantified fungal taxa by function. Figure 9 shows the community functional composition and abundance of intestinal fungi of different winged BPHs under the condition of relative abundance greater than 0.01%. The gut fungi of different winged BPHs can be divided into 3 nutritional patterns and 14 ecological functional groups with only Probable and Highly Probable confidence levels presented (Table 4).

**Table 4:** Functional classification and relative abundance of gut fungi of different winged BPHs.

Nutritional type	Functional classification	Relative abundance		Confidence level
		cdlw (%)	cdsw (%)	
Pathogenic	*Animal pathogens*	0.63	/	Probable
	*Phytopathogens*	0.11	0.03	Probable
	*total*	0.74	0.03	
Saprotrophic	*Lignicolous saprotrophs*	0.11	0.03	Highly probable
	*Coprophilous fungi*	/	0.02	Highly probable
	*Soil saprotrophs*	/	–	Probable
	*Coprophilous—soil saprotrophs*	/	0.04	Probable
	*Unclassified saprotrophs*	24.10	1.70	Probable
	total	24.21	1.79	
Symbiotic	*Ectomycorrhizal fungi*	/	0.04	Highly probable
	total	/	0.04	
Pathogenic-saprotrophic	*Animal pathogens*—*Unclassified saprotrophs*	0.03	0.04	Probable
	*Phytopathogens*—*Unclassified saprotrophs*	0.05	0.02	Probable
	total	0.08	0.06	
Pathogenic-symbiotic	*Ericoid mycorrhizal fungi*	/	0.01	Probable
	*Fungal parasite cluster—Phytopathogens*	/	0.01	Probable
	total	/	0.02	
Saprotrophic-symbiotic	*Animal symbionts—Unclassified saprotrophs*	/	0.09	Highly probable
	*Fungal parasite cluster—Soil saprotrophs*	/	0.03	Probable
	total	/	0.12	

“–” denotes trophic types and functional groups with relative abundances < 0.01% at the Probable and Highly Probable confidence levels. “/” denotes trophic types and functional groups with relative abundances 0.00% at the Probable and Highly Probable confidence levels; cdlw represents gut samples of long-winged BPHs, cdsw represents gut samples of short-winged BPH.

The results showed that among the 63 OTUs in the gut of long-winged BPH, 17 OTUs were divided into 6 fungal functional groups, accounting for about 27.00% of the total number of OTUs; *Saprotroph* is the main trophic group and *Undefined Saprotroph* is the main functional group. In addition, the 17 OTUs involved 2 phyla (*Ascomycota*, *Basidiomycota*) and 11 genera (*Talaromyces*, *Simplicillium*, *Phlebia Exobasidium*, *Uwebraunia*, unclassified_Sclerotiniaceae, *Neurospora*, *Zymoseptoria*, *Malassezia*, unclassified_Mycosphaerellaceae, *Typhula*), The most abundant are *Talaromyces* and *Simplicillium.* Among the 165 OTUs in the gut of short-winged BPH, 35 OTUs were divided into 13 fungal functional groups, accounting for about 21.21% of the total number of OTUs; Saprotroph is the main trophic type, and Undefined Saprotroph is the main functional group. And the 35 OTUs involved 3 phyla (*Ascomycota*, *Basidiomycota*, *Zygomycota*) and 31 genera (*Talaromyces*, *Saccharomyces*, *Ochroconis*, *Phaeococcomyces*, *Pichia*, *Cephaliophora*, *Malassezia*, *Trechispora*, *Phialemonium*, *Bifiguratus*, *Myrmecridium*, *Clavaria*, *Pseudeurotium*, *unclassified_Mycosphaerellaceae*, *Bryochiton*, *Suillus*, *Oidiodendron*, *Geoglossum*, *unclassified_Lasiosphaeriaceae*, *Colletotrichum*, *Wallemia*, *Piloderma*, *Cercophora*, *Russula*, *Zopfiella*, *Marasmiellus*, *Corynespora*, *unclassified_Magnaporthaceae*, *Alnicola*, *Archaeorhizomyces*, *and unclassified_Ustilaginaceae*), *Talaromyces* and *Saccharomyces* have higher abundance. The results preliminarily indicated that the gut symbiotic fungi of BPHs may have an important influence on the wing dimorphism.

### Changes in dominant gut symbionts at the genus level of different winged BPHs

**Figure 10:**
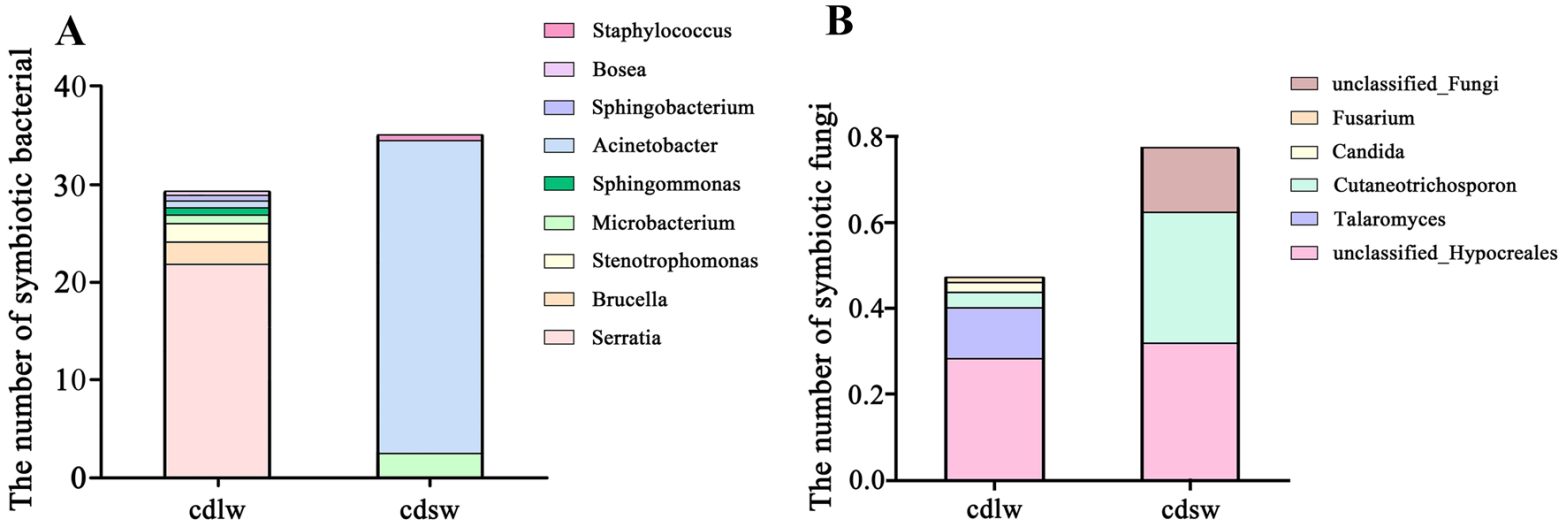
(**A**) The number of dominant symbiotic bacterial at the genus level of different winged BPHs. Dominant bacterial in the gut; (**B**) Dominant fungi in the gut; cdlw represents gut samples of long-winged BPHs, cdsw represents gut samples of short-winged BPH.

The differences in the number of dominant symbionts in the guts of different winged BPHs at the genus level are shown in the Fig. 10. Although total dominant symbiotic bacteria proved higher in the guts of short-winged BPH, at the level of individual genus, the absolute numbers of *Serratia*, *Brucella*, *Stenotrophomonas*, *Sphingomonas*, *Sphingobacterium*, and *Bosea* were far greater than those of short-winged BPH (Fig. 10A). Only *Microbacterium*, *Staphylococcus*, and *Acinetobacter* exhibited greater absolute quantities in short-winged BPH versus long-winged BPH. *Serratia* predominated in the guts of long-winged BPH, while *Acinetobacter* dominated in short-winged BPH guts (Fig. 10A). Similarly, there are differences in the number of dominant symbiotic fungi at the genus level in the guts of different winged BPHs (Fig. 10B). While total symbiotic fungi proved higher in short-winged BPH guts, analysis of individual genera revealed greater absolute quantities of *Talaromyces*, *Candida*, and *Fusarium* in long-winged BPH. In contrast, populations of *unclassified_Hypocreales*, *Cutaneotrichosporon*, and *unclassified*_*Fungi* predominated in short-winged BPH guts. Combined *unclassified_Hypocreales* and *Talaromyces* constituted the top fungal taxa in long-winged BPH guts, while unclassified_*Hypocreales* and *Cutaneotrichosporon* predominated in short-winged BPH guts (Fig. 10B).

## Discussion

There are rich symbiotic bacteria contained in the gut of BPHs can not only provide the host with nutrition that is lacking in its food or cannot be obtained in its food, but also improve the immune activity^4,26^. It can also affect the reproductive ability of the host and the resistance to insecticides and detoxification^9,12^. Therefore, it is of great significance to study the gut commensal bacteria of different winged BPHs. In this paper, by measuring the total number of symbiotic bacteria in different winged and different developmental stages of BPHs, the changes in the number of symbiotic microbiota in different winged at different developmental stages were found: The trend in symbiotic bacteria per gut unit decreased initially followed by an increase from the 1st day to the 4th day of emergence, this transient decline potentially results from individual growth outpacing symbiont proliferation, especially the short-winged BPH requires greater nutrients for reproduction; The trend in symbiotic fungi per gut unit exhibited a gradual increase from the 1st day to the 4th day of emergence, especially in the adult stage. It is speculated that the gut may contain symbiotic fungi related to the growth, reproduction and development of BPH. Brown planthopper is a serious destructive monophagous rice pest, which is rich in symbiotic microbiota^27^. Current studies on insect wing development generally believe that genetic genes and environmental factors affect insect wing shape^28^, and the role of wing type differentiation and symbionts in the body is still unknown. According to the Ace index, Chao index, Shannon index and Simpson index, the species richness of gut bacteria in short-winged BPH is higher than that in long-winged BPH, and the species diversity of gut bacteria in long-winged BPH is significantly higher than that in short-winged BPH; while the species richness and diversity of gut fungi in short-winged BPH are higher than those in long-winged BPH. Further determining the community structure and composition of symbionts in different winged BPHs and preliminarily judging the functions of symbionts populations can provide a theoretical basis for in-depth understanding of the impact of symbionts on the physiology and biochemistry of BPHs with different wing types. According to COG function prediction, in the gut bacterial samples of different winged BPHs, the metabolic pathways mainly involved in the gut bacteria of long- and short-winged BPHs are amino acid transport and metabolism, and the relative abundance of short-winged BPH bacteria is higher than that of long-winged BPH bacteria. In addition, the abundance of extracellular structures in long-winged BPH bacteria is higher than that in short-winged BPH bacteria. A variety of dominant symbiotic bacteria can degrade petroleum hydrocarbons, nitrobenzene, phosphorus and other organic matter and cellulose^29^, and are naturally resistant to a variety of antibiotics and metabolize toxic substances^30,31^. According to FUNGuild functional prediction, in the gut fungi samples of different winged BPHs, *saprotroph* is the main trophic type, and *undefined saprotroph* is the main functional group. Studies have shown that saprotrophic fungi are involved in the degradation of organic matter^32^. It is speculated that symbiotic fungi may be related to the detoxification and pesticide resistance of BPHs. The relative abundance of symbiotic fungi of long-winged BPH is higher than that of short-winged BPH, which may indicate that when the environment is harsh, the wing type differentiation of BPHs tends to be long-winged type. This study can provide a theoretical basis for further studying the regulation of symbionts of different winged BPHs on their ecological adaptability.

Combined with the results of qPCR and high-throughput sequencing, it was further verified that there are differences in the number of dominant symbiotic bacteria and fungi in the gut of different winged BPHs at the genus level. For the dominant symbiotic bacteria: the absolute number of *Serratia* in the gut of long-winged BPHs is much larger than that of short-winged BPHs. Xu et al. found that when BPHs host on resistant rice, the content of *Serratia* in the body will increase and replace *Chryseobacter* to become the dominant genus, and it is believed that *Serratia* may be related to the virulence variation of BPHs^30^. It is speculated that the rich *Serratia* in the gut of the long-winged BPH can help it adapt to the harsh environment. However, the absolute number of Acinetobacter in the gut of short-winged BPHs was greater than that of long-winged BPHs. Acinetobacter can effectively degrade cellulose, which is beneficial to providing nutrients for the reproduction and development of short-winged BPH^32^. In addition to participating in the synthesis of nutrients, it can also degrade harmful substances such as polystyrene and phenol. It is more distributed in the gut than in reproductive tissues^31^, helping the short-winged BPH to improve its detoxification ability in harsh environments and adapt to environmental changes at any time. Likewise, the number of dominant symbiotic fungi is also different. The absolute number of *Candida* in the gut of the long-winged BPH is much larger than that of the short-winged BPH. *Candida* can provide amino acids, sterols and other substances for the host and can produce a variety of detoxification enzymes to make the BPHs immune to insecticides, mycotoxins and phytotoxins^33^, which is more conducive to the BPHs from being interfered by external toxic substances during their migration in search of a suitable living environment and providing nutrients for the BPHs. The absolute numbers of *unclassified_Hypocreales* in the gut of short-winged BPH is greater than those of long-winged BPH, and the change trend was consistent with the relative abundance. Yeast-like symbionts (YLS) are common in planthoppers, which can use glutamine and other raw materials for the synthesis of essential amino acids^34^, so as to supplement the BPH with amino acids that are missing in food or cannot be synthesized by itself, and the difference in nutrients can affect the difference in wing type differentiation. It is speculated that symbiotic fungi directly or indirectly affect the wing type differentiation of BPH.

The research enriches the theory of wing type differentiation and the role of symbionts in vivo, it preliminary indicates that the gut symbionts may have an important influence on the wing type differentiation of BPH. However, due to the variety of symbiotic microbiota and the difficulty of isolation, the specific effects of symbionts on different winged BPHs need to be further studied.

## Conclusions

In summary, we investigated symbiotic microbiota in the gut of female BPHs. Based on fluorescent quantitative PCR and high-throughput sequencing and other technologies, we uncovered divergences in symbiont quantities, taxa, community structures, and dominant populations across developmental stages and wing morphs in BPHs. These findings hold significance for elucidating the topical area of BPH-endosymbiont interplay and could furnish novel perspectives on brown planthopper control from a microscopic perspective.

### Supplementary Information

Supplementary Figures.

## Data Availability

Illumina sequencing reads from N. lugens gut were submitted to the NCBI Genbank under accession number: SAMN38747257, SAMN38747258, SAMN38747259, SAMN38747260, SAMN38747261, SAMN38747262, SAMN38747263, SAMN38747264, SAMN38747265, SAMN38747266, SAMN38747267, SAMN38747268, SAMN37988708, SAMN37988710, SAMN37988697, SAMN37988582, SAMN37988448, SAMN37987560, SAMN37934494, SAMN37934487, SAMN37934485, SAMN37934484, SAMN37934473, SAMN37933095.
