# Supplementary material for: Study on the gut symbiotic microbiota in long- and short-winged brown planthopper, Nilaparvata lugens (Stål)
Source: Sci Rep. 2024 May 17;14:11306. doi: 10.1038/s41598-024-62350-2 (PMC11101650; doi:10.1038/s41598-024-62350-2)
Supplement: Supplementary file 1 — Supplementary Figures. [file 41598_2024_62350_MOESM1_ESM.docx]

The diversity index box diagram of symbionts of different winged types BPHs at the OTU level is as follows :


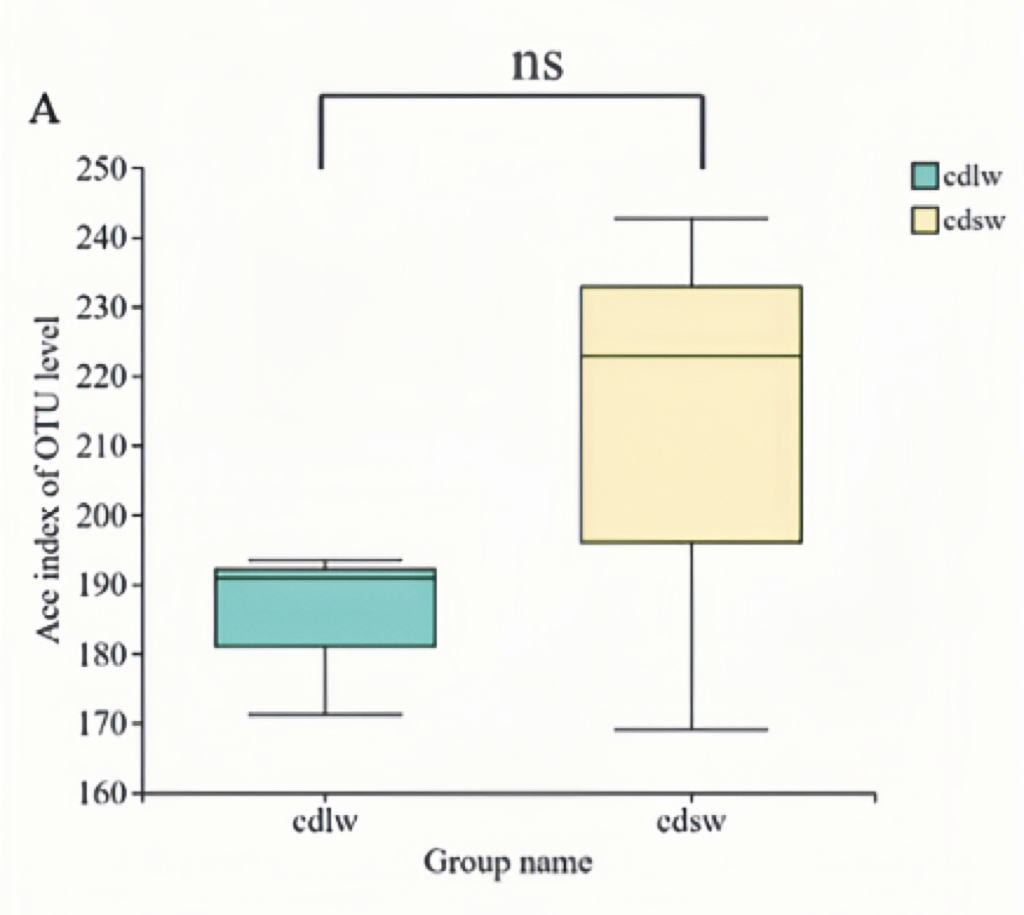

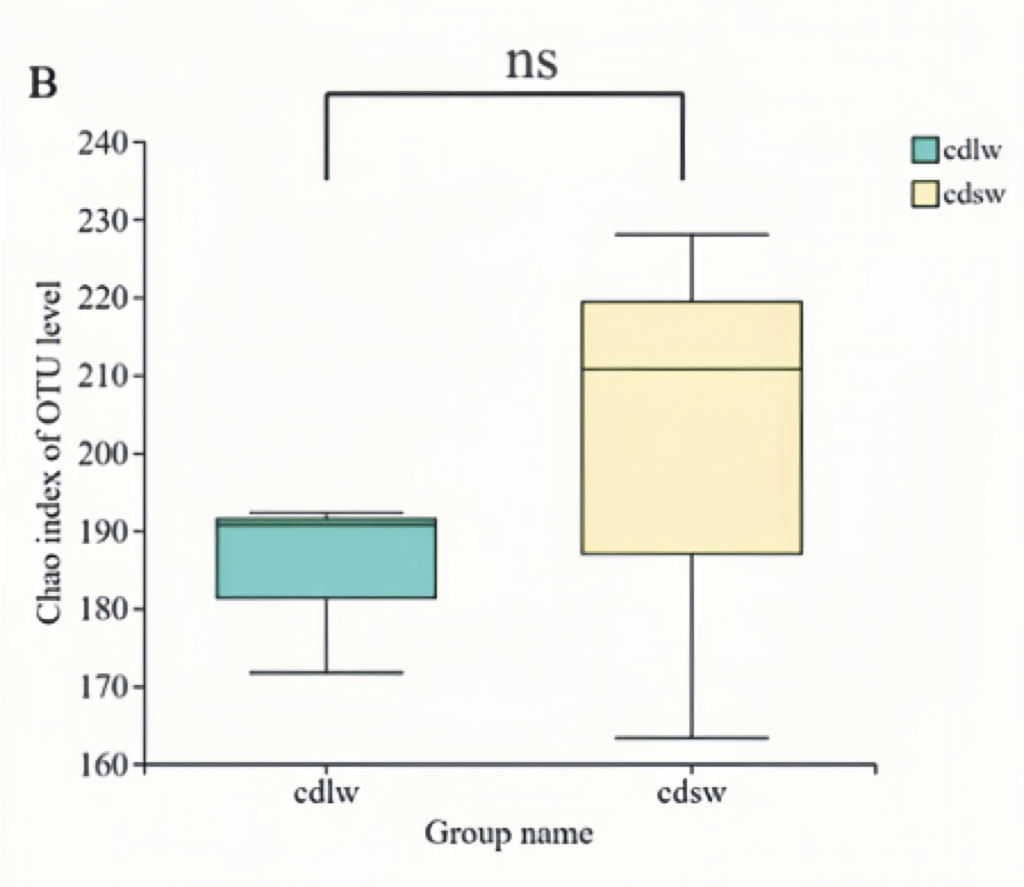

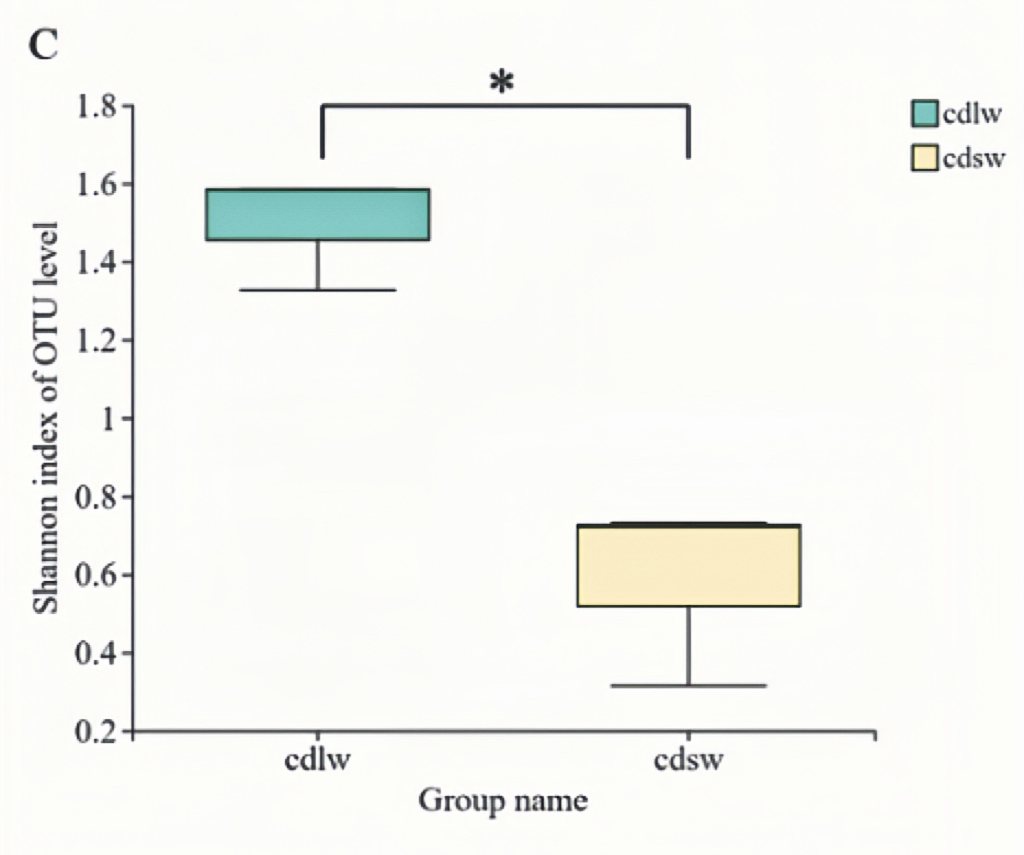

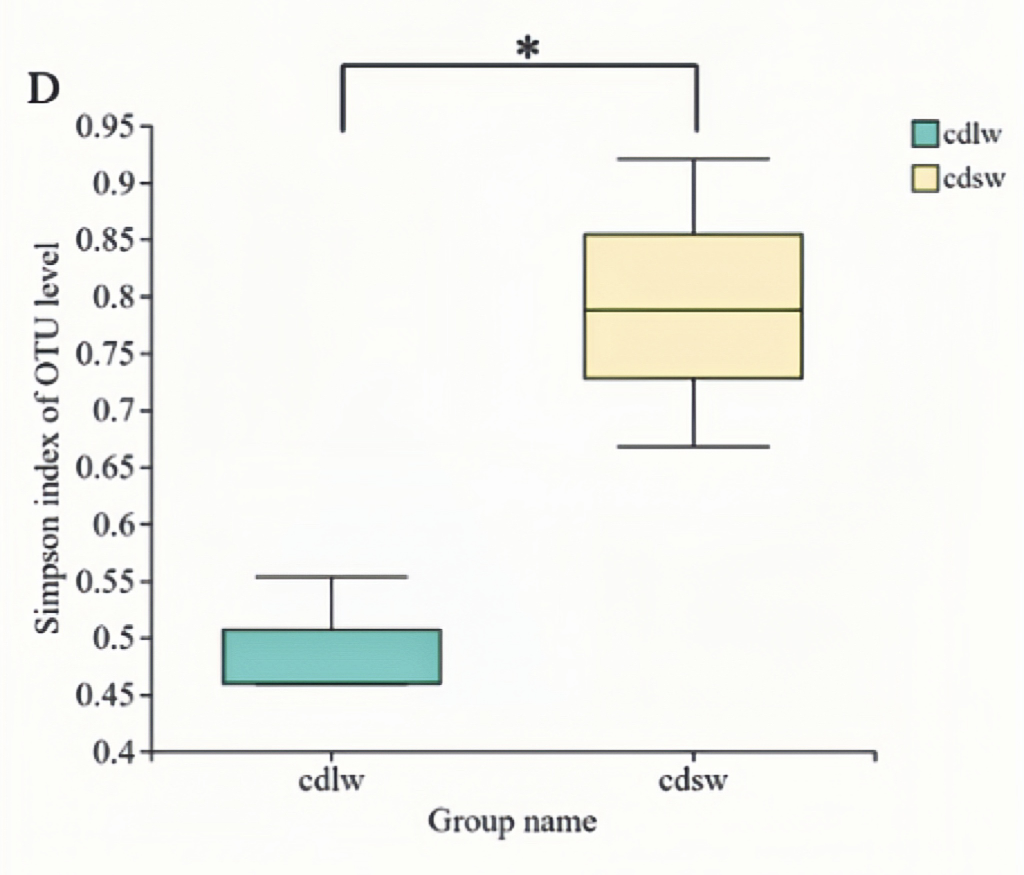


Fig1.（A-D）The diversity index box diagram of gut symbiotic bacteria of different winged BPHs at OTU level, cdlw represents the gut samples of long-winged BPH ; cdsw represents the gut samples of short-winged BPH.


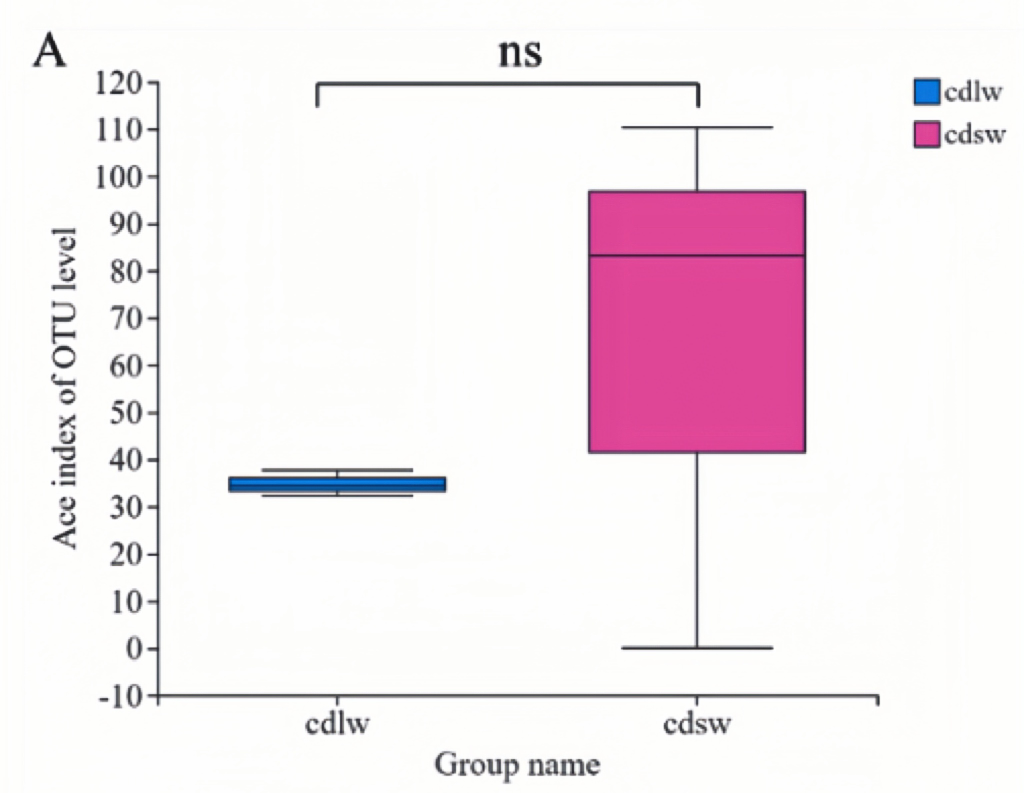

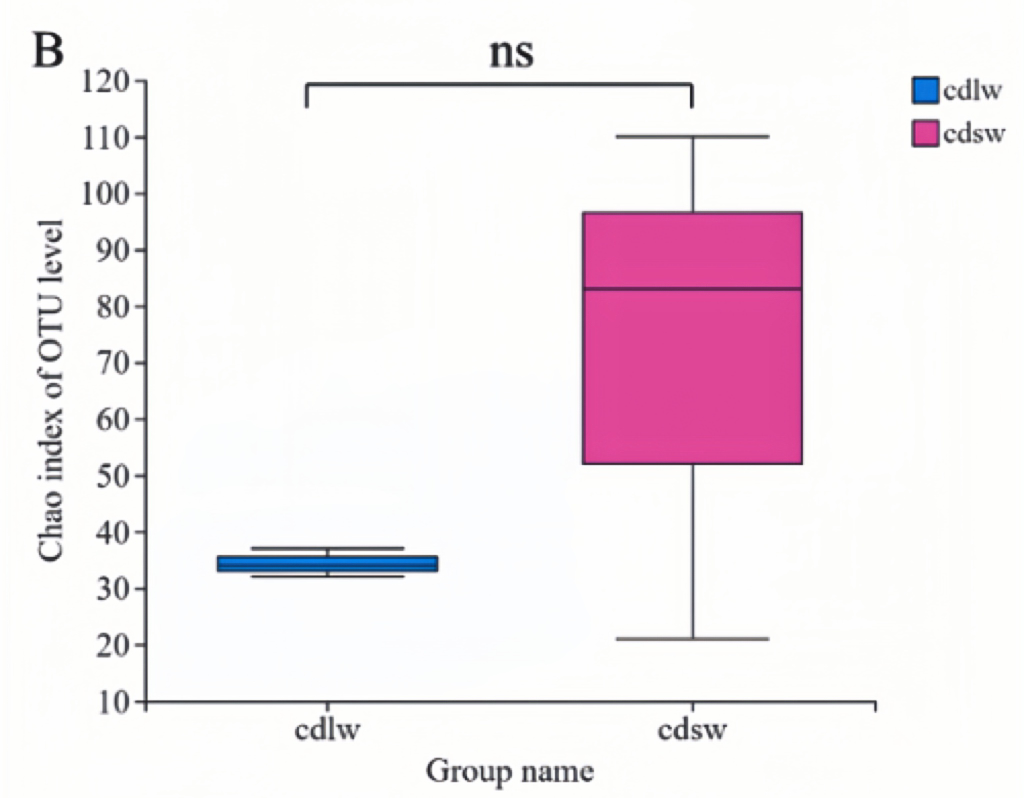

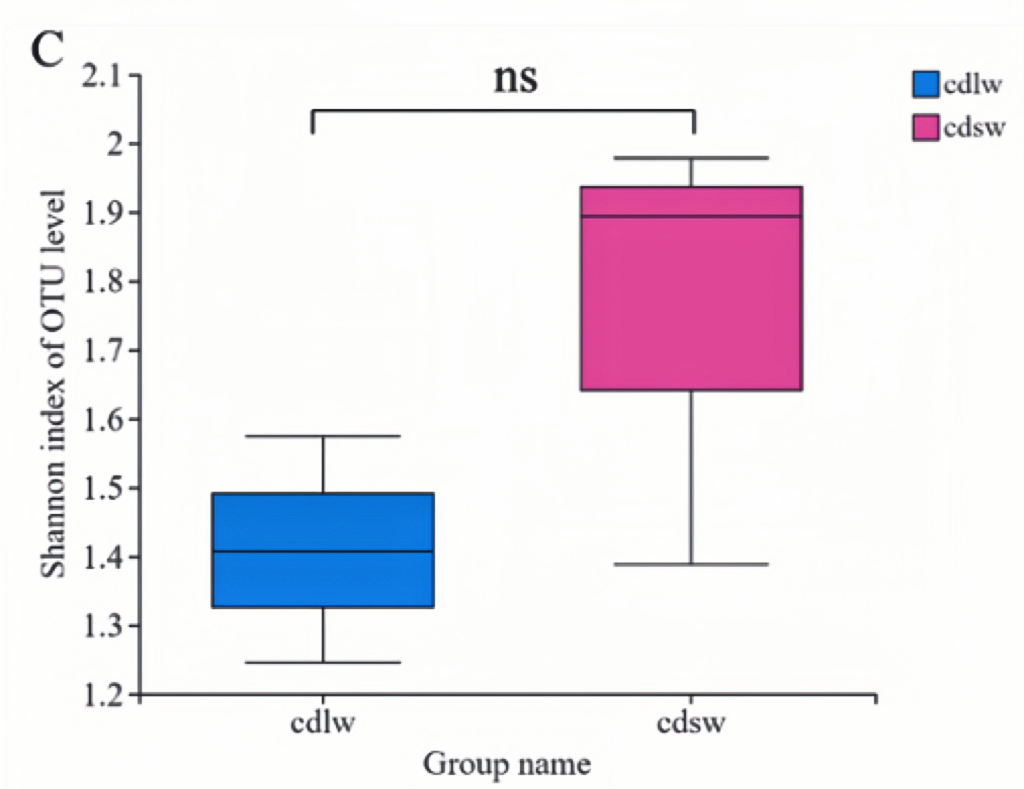

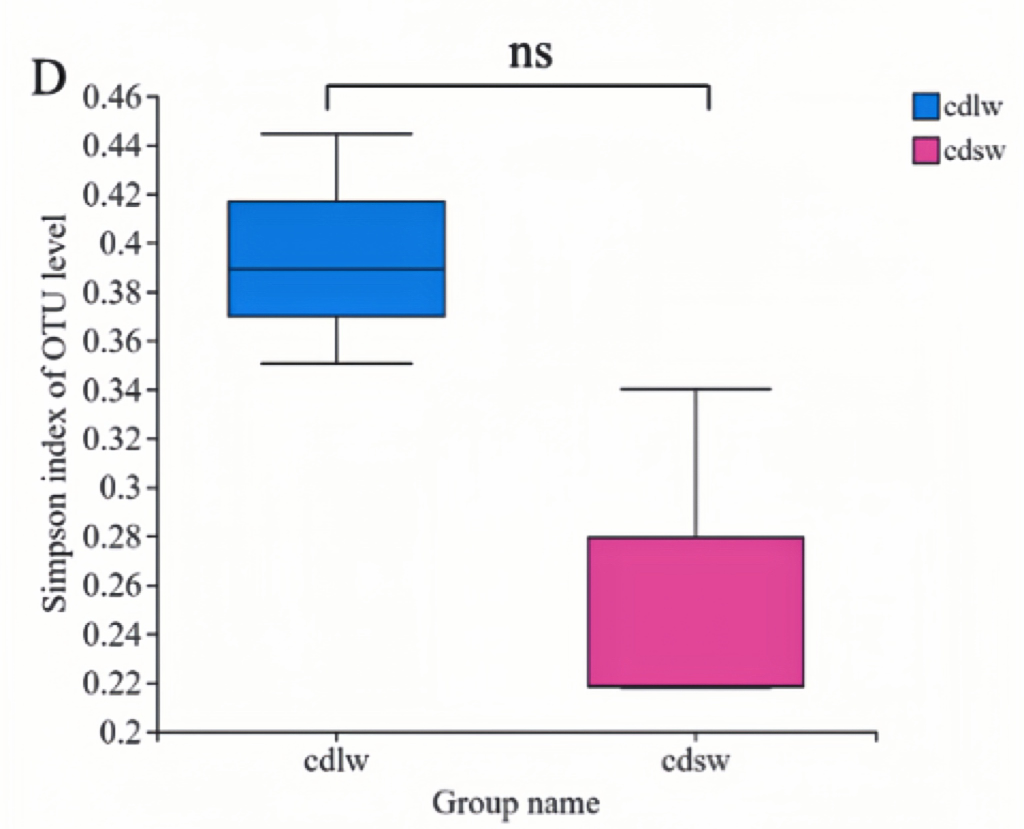


Fig2.（A-D）The diversity index box diagram of gut symbiotic fungi of different winged BPHs at OTU level, cdlw represents the gut samples of long-winged BPH; cdsw represents the gut samples of short-winged BPH.


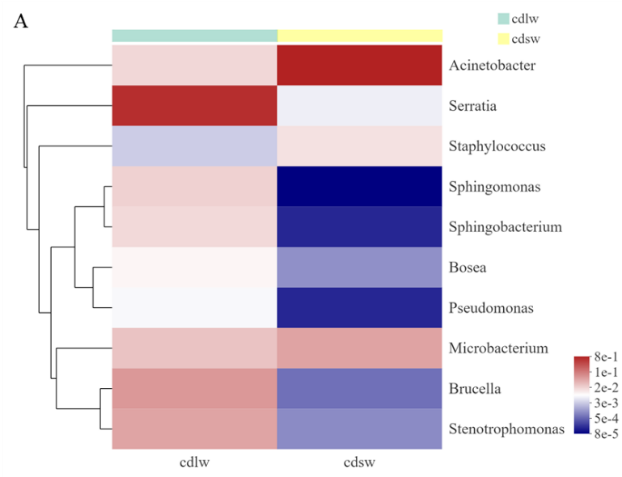


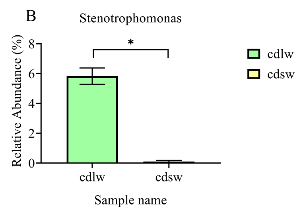

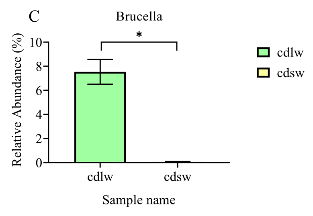

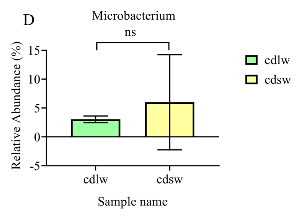

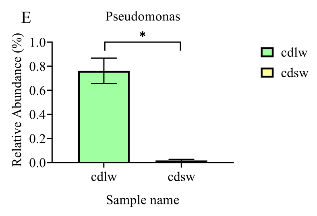

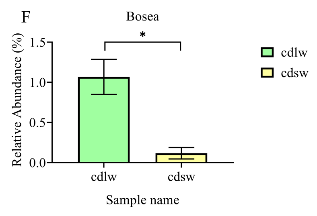

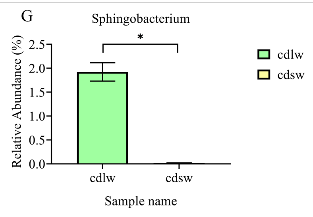

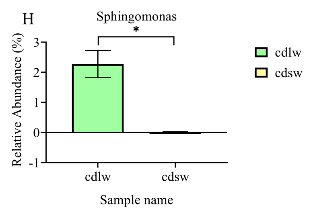

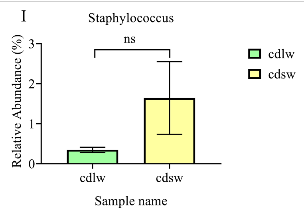

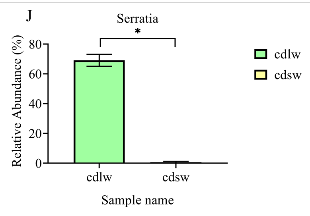

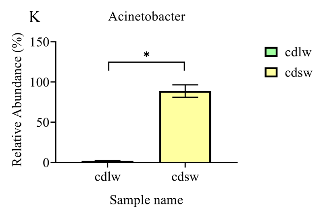


Fig.3 Difference analysis of gut dominant bacteria in different winged BPHs at the genus level. (A) Heatmap diagram of gut symbiotic bacteria in different winged BPHs; ( B-K ) the difference analysis of the relative abundance of gut symbiotic bacteria in different winged BPHs.

Note: cdlw represents the gut sample of long-winged BPH, and cdsw represents the gut sample of short-winged BPH. ' * ' indicated that there was a significant difference in the genus level of gut symbiotic bacteria among different winged BPHs( P < 0.05 ). ' ns ' indicated that there was no significant difference in gut symbiotic bacteria among different winged BPHs. Welch T test was used for significant difference analysis.


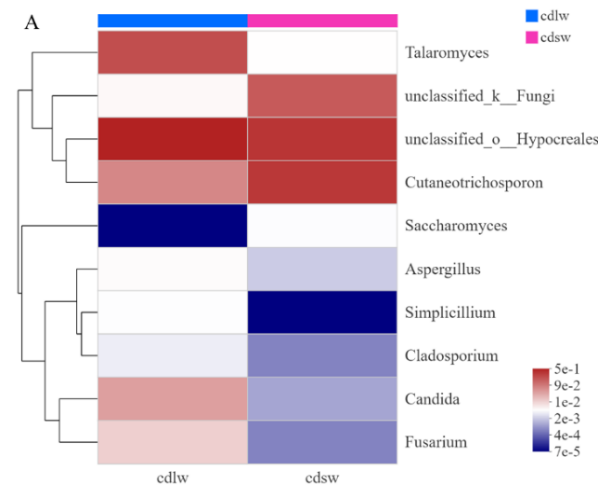


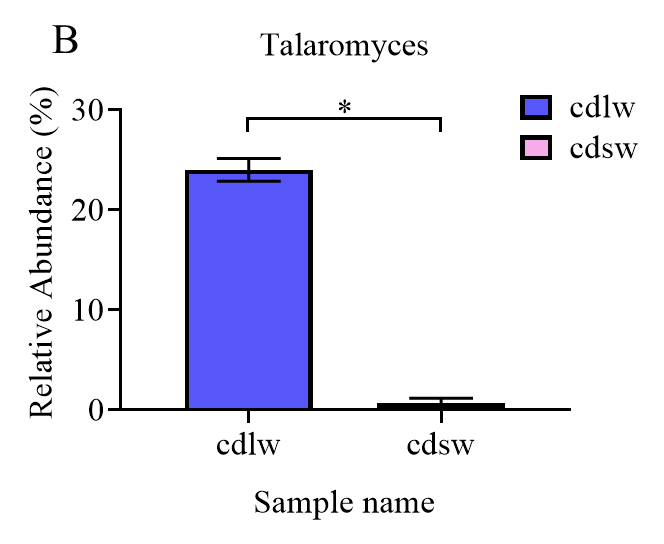

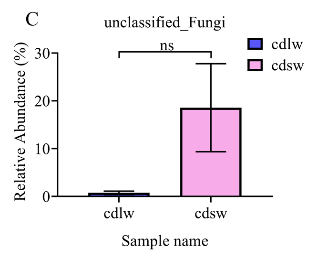

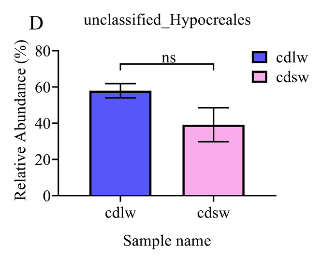


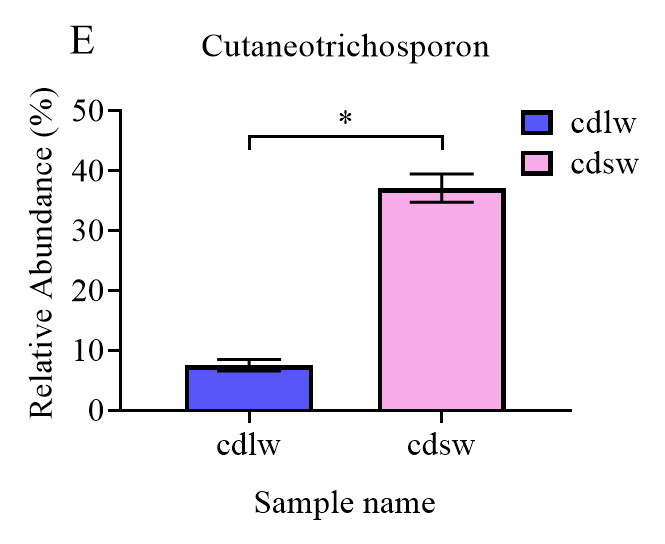

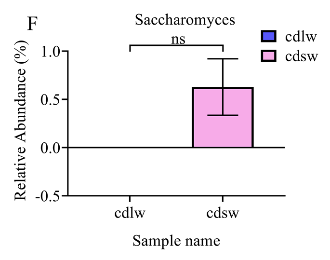

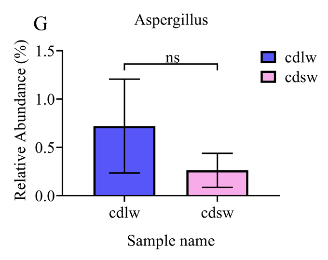


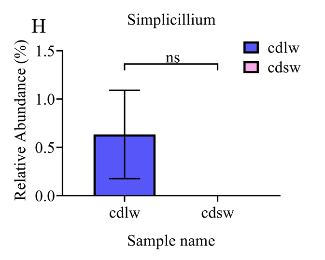

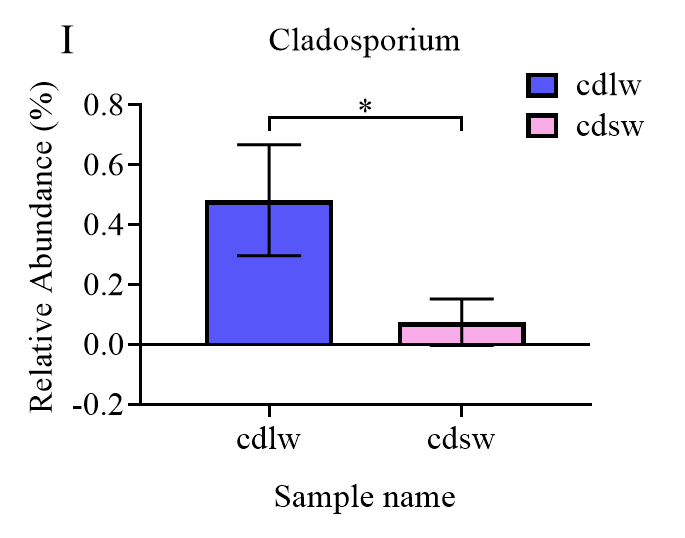

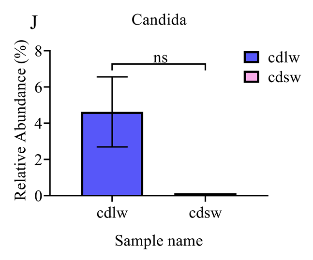


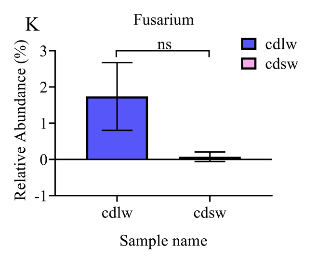


Fig.4 Difference analysis of gut dominant fungi in different winged BPHs at the genus level. (A) Heatmap diagram of gut symbiotic fungi in different winged BPHs; ( B-K ) the difference analysis of the relative abundance of gut symbiotic fungi in different winged BPHs.

Note: cdlw represents the gut sample of long-winged BPH, and cdsw represents the gut sample of short-winged BPH. ' * ' indicated that there was a significant difference in the genus level of gut symbiotic fungi among different winged BPHs ( P < 0.05 ). ' ns ' indicated that there was no significant difference in gut symbiotic fungi among different winged BPHs. Welch T test was used for significant difference analysis.
